# Atherosclerosis and Alzheimer - diseases with a common cause? Inflammation, oxysterols, vasculature

**DOI:** 10.1186/1471-2318-14-36

**Published:** 2014-03-21

**Authors:** Richard Lathe, Alexandra Sapronova, Yuri Kotelevtsev

**Affiliations:** 1 State University of Pushchino, Prospekt Nauki, Pushchino 142290, Moscow Region, Russia; 2 Pushchino Branch of the Institute of Bioorganic Chemistry, Russian Academy of Sciences, Pushchino 142290 Moscow Region, Russia; 3 Pieta Research, PO Box 27069, Edinburgh EH10 5YW, UK; 4 Optical Research Group, Laboratory of Evolutionary Biophysics of Development, Institute of Developmental Biology of the Russian Academy of Sciences, Moscow, Russia; 5 Biomedical Centre for Research Education and Innovation (CREI), Skolkovo Institute of Science and Technology, Skolkovo 143025, Russia; 6 Centre for Cardiovascular Science, Queens Medical Research Institute, University of Edinburgh, Little France, Edinburgh EH16 4TJ, UK

**Keywords:** Atherosclerosis, Alzheimer, APOE, Infection, Inflammation, Cholesterol, 25-hydroxycholesterol

## Abstract

**Background:**

Aging is accompanied by increasing vulnerability to pathologies such as atherosclerosis (ATH) and Alzheimer disease (AD). Are these different pathologies, or different presentations with a similar underlying pathoetiology?

**Discussion:**

Both ATH and AD involve inflammation, macrophage infiltration, and occlusion of the vasculature. Allelic variants in common genes including *APOE* predispose to both diseases. In both there is strong evidence of disease association with viral and bacterial pathogens including herpes simplex and *Chlamydophila*. Furthermore, ablation of components of the immune system (or of bone marrow-derived macrophages alone) in animal models restricts disease development in both cases, arguing that both are accentuated by inflammatory/immune pathways. We discuss that amyloid β, a distinguishing feature of AD, also plays a key role in ATH. Several drugs, at least in mouse models, are effective in preventing the development of both ATH and AD. Given similar age-dependence, genetic underpinnings, involvement of the vasculature, association with infection, Aβ involvement, the central role of macrophages, and drug overlap, we conclude that the two conditions reflect different manifestations of a common pathoetiology.

**Mechanism:**

Infection and inflammation selectively induce the expression of cholesterol 25-hydroxylase (CH25H). Acutely, the production of ‘immunosterol’ 25-hydroxycholesterol (25OHC) defends against enveloped viruses. We present evidence that chronic macrophage CH25H upregulation leads to catalyzed esterification of sterols via 25OHC-driven allosteric activation of ACAT (acyl-CoA cholesterol acyltransferase/SOAT), intracellular accumulation of cholesteryl esters and lipid droplets, vascular occlusion, and overt disease.

**Summary:**

We postulate that AD and ATH are both caused by chronic immunologic challenge that induces CH25H expression and protection against particular infectious agents, but at the expense of longer-term pathology.

## Background

Better nutrition and lifestyle changes make important contributions to extending human lifespan, but new morbidities are encountered with aging, notably AD and ATH. At first sight these appear to be different conditions. In the present debate we address whether the two conditions are different, or instead share a common etiology. We build upon a previous debate – *Ill or Just Old?* – and agree with Izaks and Westendorp that ‘we should investigate the risk factors (component causes) of diseases in the latter part of life’ [1]. The discussion here commences with age-related risk factors, genetic predispositions, animal models, and the central involvement of the vasculature and inflammation. We then extend the discussion to infection, amyloid β, animal models, infection, drugs, and the central signaling role of cholesterol derivatives. We suggest that both conditions result from an inflammatory disorder as a result of an infectious condition, both crucially linked to sterol metabolism and innate immunity, leading to vascular occlusion.

## Discussion

### Disease characteristics

**Figure 1:**
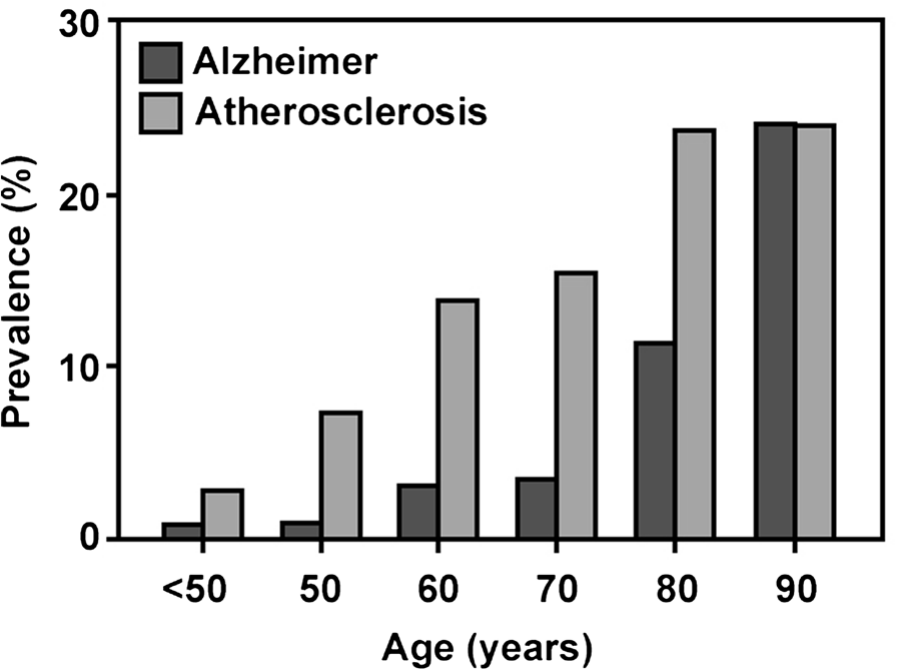
**Age-dependence of Alzheimer disease and atherosclerotic vascular disease.** Compiled from multiple sources including [2-5].

AD is the main form of dementia (~70%) in Western countries, and is characterized by the presence in postmortem brain of extracellular amyloid plaques composed of ‘Aβ’ generated by the aggregation of toxic peptide fragments of the Alzheimer precursor protein, APP, and intraneuronal deposition of highly phosphorylated filamentous aggregates (neurofibrillary tangles, NFT) of the microtubule-associated protein Tau. Onset is typically above age 70 (Figure 1).

By contrast, ATH (from Greek *athera*, ‘gruel’: gruel-like deposits, and *sclerosis*, ‘hardening’), also known as arteriosclerotic vascular disease, is not a unitary disorder, and instead ranges from primary arterial atheroma – inflammation and accumulation of cholesterol-laden macrophages in the walls of major arteries – to ‘plaque’ formation and inflammation in the arterial wall [6], leading to progressive occlusion, with consequent risk of myocardial infarction or cerebral stroke because plaque rupture can provoke thrombosis. Disease development is accompanied by disruption of the endothelial cell layer, vascular smooth muscle cell migration, and matrix calcification. Onset is a little earlier than for AD, but ultimately affects a similar proportion of the elderly (Figure 1).

The ongoing rise in both AD and ATH has been ascribed, rightly or wrongly, to the increasing adoption of a Western sedentary lifestyle accompanied by a diet rich in fats and sugars. Both disorders are essentially unknown in children and young adults, with onset in later life (Figure 1).

### Vascular involvement

At first glance the two diseases would appear to be distinct, with ATH being characterized by cholesterol-rich deposits in arterial walls and AD by neuronal loss, NFT, and amyloid plaque formation. However, there is increasing evidence that AD is also associated with vascular dysfunction. Although the structure of cerebral arteries and arterioles differs somewhat from that of the major blood vessels, they are similarly dependent on endothelial and smooth muscle cells [7].

Studies in AD mouse models have confirmed that disease development is associated with deposits in the cerebral arterial vasculature [8]. In patients, extracellular deposits of amyloid in AD brain are principally associated with the cerebral arterial vasculature, and deposit density declines with distance from the larger vessels [9,10]. It has been postulated that dysfunction of vascular endothelial cells lining brain blood vessels plays a central role in precipitating neuronal death [11].

**Figure 2:**
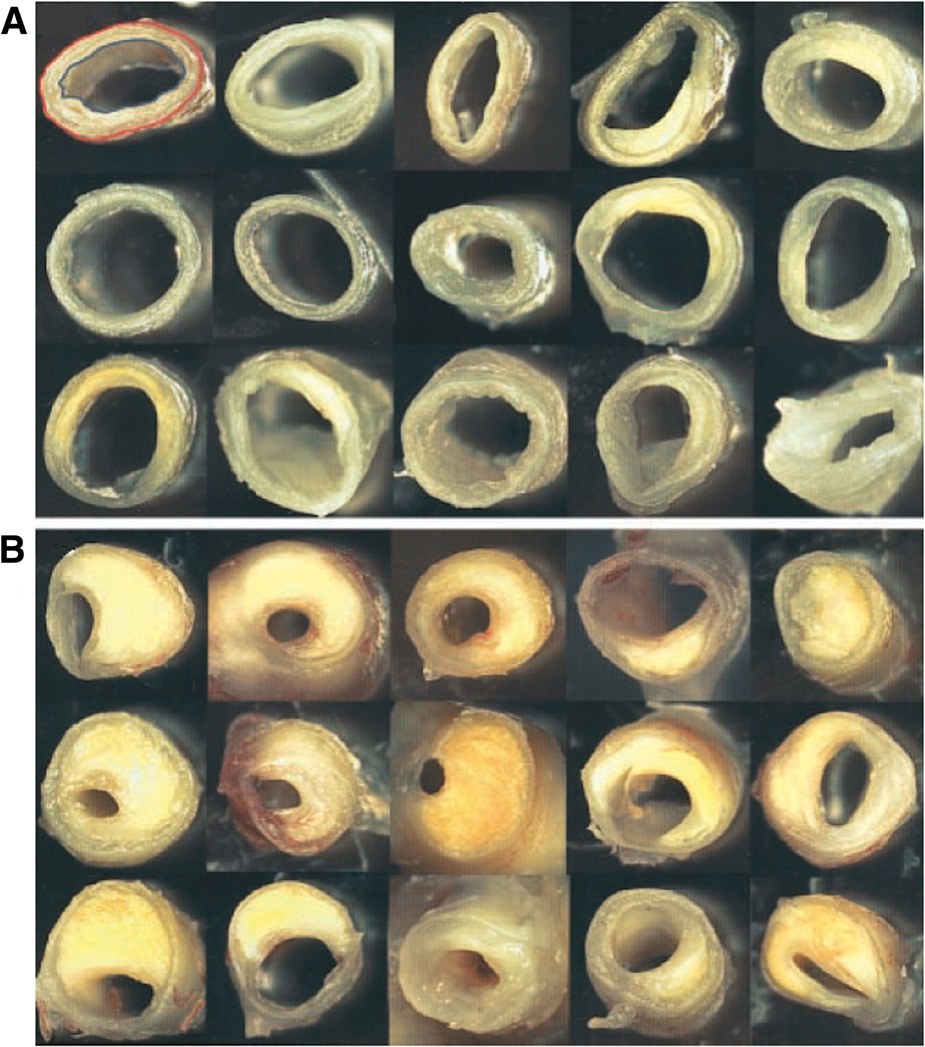
**Occlusions of brain blood vessels (‘circle of Willis’) in controls and AD.** Panel **(A)** shows cerebral arteries from non-demented elderly individuals, whereas Panel **(B)** shows arteries from AD patients showing atheromatous plaque deposition. Figure reproduced, with permission, from [15].

Brain scanning revealed that AD is associated with decreased cerebral blood flow [12,13], as also seen in AD mouse models [14]. Roher [15] examined cerebral arteries from confirmed AD cases and age-matched non-demented controls. In addition to plaques and tangles, it was found that AD cases displayed a degree of cerebral artery (circle of Willis) occlusion that was significantly greater than in controls (Figure 2), and there was a positive correlation between the degree of arterial stenosis and NFT score [15]. This finding was confirmed in a study by Hofman *et al.* who examined AD patients and controls for markers of atherosclerosis including vessel wall thickness as assessed by ultrasonography. All markers of ATH were over-represented in AD patients versus controls, and the odds ratio for AD in those with significant ATH versus those without was 3.0 (CI 1.5–6) [16]. Since then the lead findings have been widely confirmed [17-19]; the link between intracranial atherosclerosis and AD is not an artifact of diagnostic misclassification [20].

The recent Baltimore Longitudinal Study of Aging (BLSA) found that individuals with (non-brain) coronary or aortic ATH *per se* are not at increased risk of AD. However, intracranial atherosclerosis was confirmed as a strong risk factor for dementia [21].

It remains possible that AD might encompass two distinct conditions: a major class with involvement of the cerebral vasculature, and a minor class in which no such involvement is apparent. However, this is unclear. Ellis *et al.* provide evidence that the major class of AD (83%) is associated with brain angiopathy [22]. The second most common classification (15%) of senile dementia, cerebral amyloid angiopathy (CAA/vascular dementia), is primarily associated with amyloid-positive lesions of the cerebral vasculature, and has substantial overlaps with both ATH and AD [23,24]. Further studies are needed on the subclassification of AD-related senile dementias according to type of vascular involvement. However, the combined evidence demonstrates that the large majority of clinically diagnosed AD cases display significant vascular involvement.

**Figure 3:**
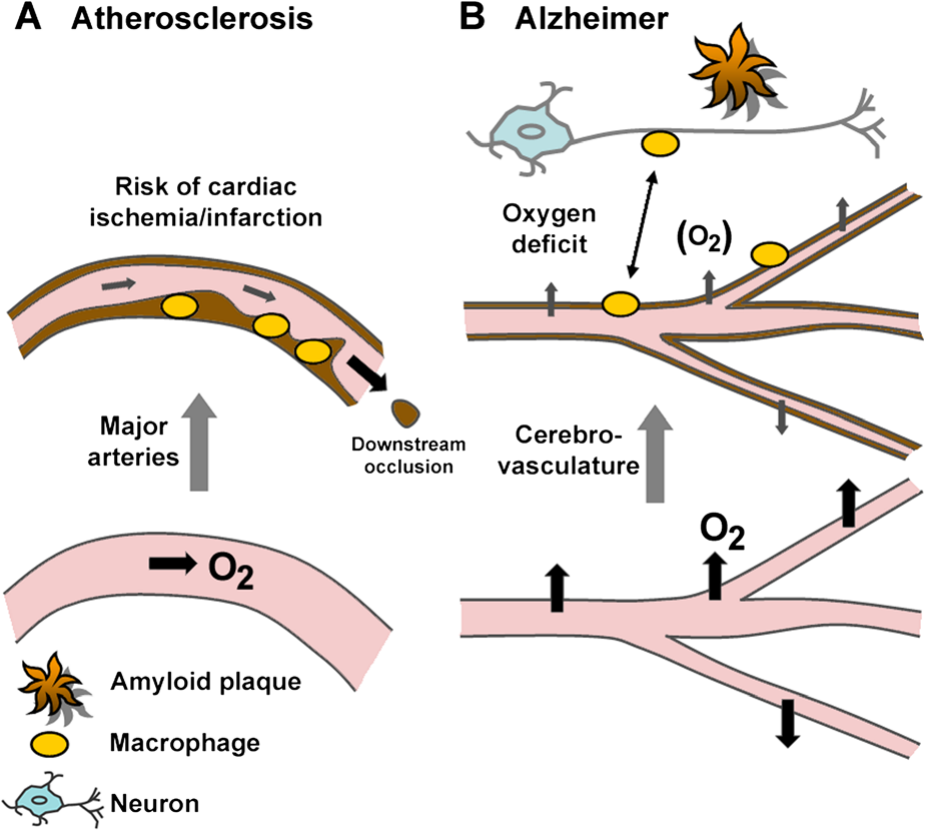
**Differential contribution of vessel wall thickening (dark-brown coloration) to disease. (A)** Atherosclerosis is a chronic inflammatory condition characterized by the accumulation of cholesterol-laden macrophages (foam cells) in arterial walls, partial occlusion and, when the plaques rupture, risk of myocardial infarction and stroke. Partial occlusion compromises oxygen supply to other tissues. **(B)** In Alzheimer disease neuronal loss is accompanied by thickening of brain vessel walls, recruitment of macrophages, and the formation of amyloid deposits of Aβ in the vicinity of the cerebrovasculature. Macrophages are implicated in shuttling Aβ between amyloid deposits and vessel walls. Mechanisms underlying neuronal loss are not understood, but impaired *trans*-vessel oxygen and glucose delivery, and reduced removal of toxic metabolites, may predispose to neuronal death; impairment of blood–brain barrier function may also contribute.

In sum, the major forms of both AD and ATH are associated with vascular wall thickening and blood vessel occlusion. The predominant localizations differ (major arteries in ATH, cerebral arterial vasculature in AD); the pathways leading to disease may also differ. In ATH, vascular deposits impair heart function and are at significant risk of entering the circulation, leading to stroke. In AD, brain hypoperfusion has been causally associated with disease [25]. We surmise that thickening of the cerebrovasculature leads to impaired O_2_ and nutrient delivery to the brain, predisposing to neuronal loss (Figure 3). These pathways are not necessarily independent: ATH alone might compromise cerebral O_2_/nutrient supply and, conversely, AD-like processes in crucial brain regions could deregulate the cardiovascular system.

Overall, the evidence suggests that AD and ATH represent a spectrum of related conditions, with vascular involvement as a common predisposing factor, although the site of vascular involvement differs between the two diseases. We next dwell on the different genetic risk factors and how they cast light on the relationship between AD and ATH.

### Genetic predisposition

#### Polymorphic loci associated with both diseases

If there is an overlap between AD and ATH it would be expected that risk alleles would be shared between the two diseases. Hyperlipidemia is a risk factor for both diseases, and mutations leading to hyperlipidemia are major risk factors for ATH (not reviewed). In AD the situation is complicated because individuals with hypercholesterolemia generally die at a younger age; but, for example, early signs of cognitive impairment are seven-fold increased in patients with hyperlipidemia due to low-density lipoprotein receptor (LDLR) mutations [26].

Polymorphisms associated with disordered lipid metabolism showing evidence of bias in both diseases include a gene cluster on chromosome 2q14-21: bridging integrator 1/amphiphysin II, *BIN1* (2q14) – cytochrome P450, family 27, subfamily C, polypeptide 1 (potential cholesterol hydroxylase), *CYP27C1* (2q14.3) – and excision repair cross-complementing repair deficiency complementation group 3, *ERCC3* (2q21), over 0.3 Mb. Genome-wide association studies (GWAS) of lipid metabolic disorders have highlighted further associations with polymorphisms in the genes cholesteryl ester transfer protein, plasma, *CETP* (16q21); clusterin, *CLU* (8p21-p12); complement component 3b/4b receptor 1, *CR1* (1q32); and low-density lipoprotein receptor, *LDLR* (19p13.2) (for reviews and databases see [27-30] and the Catalog of Published Genome-Wide Studies; http://www.genome.gov/gwastudies/).

However, interpreting the link between genes and disease is difficult because (i) GWAS studies require polymorphisms in the population, and genes lacking polymorphisms are therefore not identified, and (ii) it is difficult to dissociate direct causality from indirect association. The exception is the gene encoding apolipoprotein E, *APOE* (19q13.2).

#### The APOE locus: one candidate gene or several?

Multiple GWAS studies have firmly highlighted alleles in and around the *APOE* locus as risk factors for both diseases (http://www.genome.gov/gwastudies/).

The *APOE* gene is located within a tight cluster of genes at chromosome 19q13: poliovirus receptor-related 2/herpesvirus entry mediator B/nectin-2, *PVRL2* (19q13.2) – translocase of outer mitochondrial membrane 40, *TOMM40* (19q13) – *APOE* (19q13.2) – apolipoproteins C-I, C-II, C-IV, *APOC1*/*C2*/*C4* (all 19q13.2) – and cleft lip and palate associated transmembrane protein 1, *CLPTM1* (19q13.3), over a distance of 0.1 Mb. Although work on *APOE* alleles has dominated the field (discussed below), linkage disequilibrium between SNPs in different genes suggests that genes other than *APOE*, notably *PVRL2*, *TOMM40*, and *APOC1*, may influence the development of AD [31]. Attention has focused on an intronic poly(T) polymorphism in different *TOMM44* alleles. Although some studies found no association between disease (AD and/or ATH) and *TOMM40* variants [32,33], others reported associations, but in opposite directions [34-36], a possible indication of population-specific risk factors. More detailed analysis [37] indicates that there are several different allelic variants in this poly(T) region, and some appear to associate with age of AD onset. Mice knocked out for another component of the TOMM complex, TOMM5, display a complex inflammatory lung phenotype [38], but possible predisposition to age-related disease was not studied. There also appears to be complex transcriptional interplay between *APOE* and *TOMM44*[39].

Overall, the role of *APOE* in both AD and ATH has been confirmed independently by multiple studies and by transgenic modeling (below), but it remains open whether linked genes, possibly *TOMM40*, also contribute to the pathoetiology of AD and/or ATH [40].

### Role of APOE

The ϵ4 allele at the *APOE* locus is a major risk factor for both diseases. APOE protein is a lipid transport molecule that circulates in the blood in a complex with lipid-rich lipoprotein particles that transport largely insoluble cholesterol. Lipoproteins, named on the basis of density (principally low-density lipoprotein, LDL; high-density lipoprotein, HDL; and very low density lipoprotein, VLDL), consist of phospholipids, cholesterol esters, and cholesterols, organized into 20–50 nm micelles with apolipoproteins at their surfaces.

Although detailed summary would be out of place here, it is generally held that LDL and VLDL mediate cholesterol transport between the liver and peripheral tissues [41-44]. The principal apolipoprotein is APOB100, and both APOB100 and APOE bind to the cellular LDL receptor to facilitate cellular uptake. APOE binding to LDLR (and the LDLR-related receptor, LRP1; the VLDL receptor, and the APOE receptor 2) thereby plays a role in cholesterol delivery. Conversely, HDL particles predominantly contain APOA1, A2, C, and E, and mediate reverse cholesterol transport from peripheral tissues to the liver for secretion into bile [45], and hepatic uptake is largely mediated by APOA1 binding to specific receptors on hepatocytes. APOE is a minor but crucial lipoprotein component in much of the body, but is the major apolipoprotein in cerebrospinal fluid.

There is a third role, where APOE mediates hepatic uptake of intestinally-derived remnant lipoproteins. Cell-surface heparan sulfate proteoglycans (HSPG) appear to function as a receptor for APOE [46]. In all three roles, APOE is likely to govern export of cholesterol from the cell, and thus the deposition of cholesterols in lipid-rich intracellular aggregates in the vascular wall. Allelic variants of APOE alter the function of the protein in several ways.

#### APOE variants in AD and ATH

There are three principal alleles at the apolipoprotein E locus, *APOE* (19q13.2): *ϵ2* (cys112, cys158), *ϵ3* (cys112, arg158), and *ϵ4* (arg112, arg158), giving six different genotypes in human populations, with some further minor variants (reviewed in [47-49]); homozygosity for *ϵ4* is the greatest risk factor for both AD and ATH, with risk ratios declining generally *ϵ4 > ϵ3 > ϵ2*.

The allelic differences affect APO structure and function. APOE protein contains two structural domains, the N-terminal receptor-binding domain, and the C-terminal lipid-binding domain, separated by a hinge region ([50] for review). Both polymorphic sites are within the domain (amino acids 1–191) that includes the receptor-binding site.

These changes affect receptor binding. APOE3 shows reduced receptor binding compared to APOE4, and APOE2 is very markedly impaired in LDLR binding (50-fold reduction), although it can still bind to HSPG for hepatic clearance of remnant lipoproteins. APOE4 protein is also more susceptible to unfolding than E3 or E2 [51]. In addition, the polymorphic forms affect lipoprotein association. Notably, APOE2 and APOE3 bind preferentially to HDL particles, whereas APOE4 binds preferentially to VLDL [52-55].

At a functional level, APOE3 promotes markedly greater cholesterol efflux than APOE4 [56-58]. In part this may reflect APOE-mediated changes in the expression of the gene ATP-binding cassette, subfamily A, member 1, *ABCA1* (9q31.1), a locus identified by GWAS. ABCA1, the key sterol transporter in many tissues, is thought to catalytically ‘flop’ sterols from one cellular membrane to another, and thus to play a crucial role in transport of sterols out of the cell, with highest activity for side-chain oxidized cholesterols [59]. APOE4 was reported to be impaired, versus APOE3, in upregulating ABCA1 expression and cholesterol efflux from lipid-laden macrophages [60]. Thus APOE4, versus APOE2/3, is likely to enhance intracellular cholesterol accumulation, a feature of ATH lesions.

Fragments of APOE, like Aβ, can be toxic. Similarly to APP, APOE undergoes cleavage, and APOE4 is more susceptible to cleavage than APOE3 [61]. The resulting fragments can cause AD-like neurotoxicity in mouse models [62] and the lipid-binding region of APOE is required for this toxicity [63]. The mechanism and relevance remain unknown.

#### APOE plays diverse regulatory roles: infection and inflammation

APOE is not a mere cholesterol transporter and is thought to play further roles in tissue repair, immunity, inflammation, and infection [48,64]. APOE polymorphisms affect not only the function of protein in cholesterol transport but also other processes including infection and immunity (below) and tissue repair. For example, APOE4 was shown to be less effective than either E2 or E3 in promoting neuronal repair [65] but the underlying mechanisms are not understood. What remains open to debate, however, is the exact biochemical process(es) influenced by the polymorphisms that impact upon the risk of ATH or AD development – and indeed whether they are similar in the two diseases or act independently. Studies in animal models are beginning to unravel potentially separable roles of APOE and co-culprits in the two diseases.

### Familial disease and transgenic models

No causal and highly-penetrant single gene mutations are known in ATH; modeling the involvement of APOE in transgenic mice has generally relied on the use of knockout mice. Mice knocked out for APOE (*Apoe*^−/−^), particularly when fed with a high-fat diet, develop atherosclerotic lesions similar to those seen in human ATH [66]. In addition, mice deficient in the APOE-binding LDL receptor (*Ldlr*^−/−^) develop ATH [67], further accentuated by humanized APOB [68], suggesting that differential APOE binding to LDLR may underlie the role of *APOE* polymorphisms in ATH development. A caveat remains, however, because it is not known whether *Apoe* knockout affects the function of neighboring genes whose transcriptional control overlaps with that of *Apoe.*

In AD, well-known (but rare) autosomal dominant mutations are known to cause familial disease. Mutations in the gene amyloid beta precursor protein, *APP* (21q21.3), encoding the precursor to Aβ peptide, are found in many cases of familial AD, notably in a Swedish pedigree that contains a double-replacement within APP protein (Lys595 to Asn plus Met596 to Leu) that facilitates disease-specific cleavage [69], leading to pathogenic production of Aβ peptide and the deposition in brain of amyloid plaques at an early age. Mutations in the genes presenilin 1, *PSEN1* (14q24.3) and presenilin 2, *PSEN2* (1q31-q42), encoding key components of the APP processing machinery, have been found in several cohorts of familial AD [30]. These findings reinforce the tight linkage between abnormal APP processing, Aβ deposition, and AD development.

Single-gene mutations of this type lend themselves to modeling in transgenic animals, and for many years AD research has dwelt on the expression, in mouse brain, of abnormal AD-associated mutant forms of APP and or PSEN1/2. Mice expressing the Swedish variant of APP [70-72] show deposition of aggregated Aβ, and learning and memory deficits. However, transgenic mice overexpressing mutant AD-related APP (*APP*^AD^ mice) are likely to reiterate only some aspects of the human disease because (i) APP mutations are rare in sporadic AD, and (ii) Aβ is unlikely to be an essential component of sporadic AD (see later), although it clearly plays a role. Nevertheless, most work in the field has employed *APP*^AD^ animals as the best available model of AD.

### Alzheimer precursor protein (APP) modulates both AD and ATH

**Figure 4:**
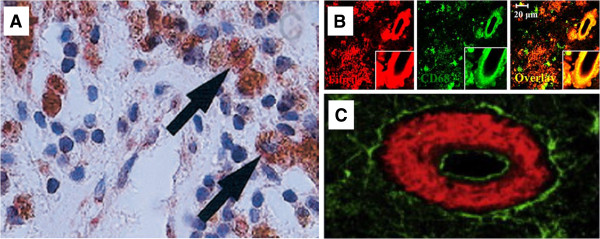
**Aβ in ATH plaque and in AD and senile cerebral amyloid angiopathy (CAA) macrophages and vessels. (A)** ATH. Colocalization of iNOS-expressing macrophages with Aβ and platelets in advanced human atherosclerotic plaque. The panel shows double immunohistochemical stain for Aβ (red) and iNOS-positive macrophages (brown), showing close colocalization (arrows). Panel adapted, with permission, from [76]. **(B)** Aβ (red) in AD neurons and perivascular macrophages (green, anti-CD68 staining, a macrophage marker). Panel adapted, with permission, from [77]**(C)** Aβ (red) in vessel walls in CAA (green staining: tissue transglutaminase, an extracellular matrix remodeling protein). Panel adapted, with permission, from [78].

AD is characterized by cerebral Aβ deposits and NFT, whereas pathologic vascular occlusion is the hallmark of ATH. However, we see again evidence of a molecular spectrum encompassing both diseases. It is notable that APOE binds to Aβ and facilitates uptake [73]; APOE4 enhances Aβ production more than APOE3, and synergizes with Aβ toxicity [74,75]. In AD, Aβ is associated with macrophages and the cerebrovasculature, notably in CAA (Figure 4), and reduced cerebral blood flow was seen in AD mice brain [14]. Macrophages ingesting Aβ have been implicated in shuttling Aβ between blood vessels and neurons [77].

APP and its toxic fragment, Aβ, are also implicated in ATH. Serum Aβ levels are reported to be elevated in stroke patients [79] and Aβ can exert toxic effects on the vascular endothelium [80,81]. Human ATH lesions have been demonstrated to contain Aβ [82] (Figure 4). Expression of AD-related APP in a strain of ATH-prone mice led to aortic atherosclerosis [83], and atherosclerotic lesions in *Apoe* knockout mice were significantly increased by overexpression of AD-related mutant APP (*APP*^AD^ mice) [84].

Conversely, genetic knockout of APP function reduced ATH plaque size by up to 90% in ATH-prone *Apoe*^−/−^ animals [85], confirming that APP plays a prominent role in ATH.

### The APOE paradox: gene knockouts reveal contrasting roles of APOE in AD and ATH models

Shared involvement of the vasculature and APP/Aβ in both ATH and AD, together with common risk loci identified by GWAS, underscores the molecular overlaps between the two conditions. Nevertheless, a central factor in both diseases is *APOE4*, and extensive studies have been carried out in transgenic and knockout mice in the attempt to unravel the molecular role of APOE.

Disease processes in both AD and ATH are accelerated in *APOE4* individuals, whereas *E2* and *E3* offer a measure of protection. However, the biochemical underpinnings that lead to disease remain obscure. One hypothesis might be that APOE4 protein accelerates uptake of cholesterol-rich particles by the vasculature, leading to more rapid disease progression. Indeed, compared to APOE4, the protective APOE2 and APOE3 proteins show reduced receptor binding.

However, genetic knockout of APOE in mice accelerates ATH [66,67], arguing against this interpretation. Similar findings were reported in mice deficient in LDLR [68], demonstrating that APOE/LDLR-mediated cholesterol export is protective against ATH.

Strikingly different observations have been made in AD models. When crossed onto an *Apoe*^*−/−*^ background no amyloid deposits were found in any brain region of transgenic *APP*^AD^ mice [86]. Vascular pathology was seen in two different lines of *APP*^AD^ transgenic mice, but when the lines were crossed to APOE knockout animals vascular Aβ pathology was abolished in both types of *APP*^AD^*Apoe*^−/−^ mice, even in very elderly animals [87]. Reduced AD-like pathology in *Apoe* knockout mice has been confirmed [88].

This result is a paradox because APOE4 confers greatest susceptibility to both disorders. It is unknown how APOE knockout can accentuate ATH in mouse models, but reduce AD development. We conclude that APOE protein function normally protects against ATH, but co-contributes to AD, suggesting a bifurcation of the pathways leading to ATH versus AD. A potential difficulty is that mice do not reiterate all aspects of either disease, and Aβ alone is not an accurate proxy for human AD. Although true human APOE deficiency is associated with marked risk of ATH [89], it remains unknown whether such deficiency in human protects against, or accelerates, AD development.

### Other gene knockouts – parallel effects on ATH and AD

Although knockout of the *Apoe* gene differentially affects disease development in ATH and AD mouse models, this was not found for other genes studied. Other knockouts generally influence disease onset/progression similarly for ATH and AD (Table 1). The role of LDLR in AD pathology remains somewhat unclear because LDLR knockout appeared not to affect disease development in one AD model [107] whereas there was a significant increase in Aβ deposition in other *APP*^AD^*Ldlr*^−/−^ mice [108], as confirmed [109], and, unlike APOE, elimination of LDLR appears to increase the severity of both AD and ATH in the relevant mouse models.

**Table 1:** Gene knockouts in mice and disease progression in ATH and AD models

**Gene**^**a**^	**ATH**	**Refs**	**AD**	**Refs**
*Abca1*	↑↑^b^	[90,91]	↑↑	[92]
*Acat1*^*c,d*^	↓↑	[93-96]	↓↓	[97]
*Acat2*^*c,d*^	↓↓	[98,99]	n/a^e^	
*Apoe*	↑↑	See text^f^	↓↓	See text^e^
*App*	↓↓	[85]; conversely, *App* overexpression amplifies ATH (see text)	↓↓*	*By inference; the production of Aβ (processing product of human APP) is central to AD pathology
*Clu1*	↓↓	[100]	↓↓↑?	[101,102]
*Cyp7b1*	↑↑	[103]	↑↑**	**By inference; *CYP7B1* expression is downregulated in AD patient brain [104]
*Ifngr1*	↓↓	[105]	↓↓	[106]
*Ldlr*	↑↑	[67,68]	↑↑	[107-109]
Other immune system components	↓↓^g^	See text	↓↓(↑)^f^	See text

^a^Gene names: *Apoe*, apolipoprotein E; *App*, amyloid beta precursor protein; *Abca1*, ATP-binding cassette, sub-family A (ABC1), member 1 (cholesterol efflux regulatory protein); *Acat1/2*, acyl-CoA cholesterol acyltransferases 1/2 [SOAT1/2; see note (d) on nomenclature]; *Clu1*, clustered mitochondria (CluA/CLU1) homolog; *Cyp7b1*, cytochrome P450 7B1 (sterol and steroid 7α-hydroxylase); *Ifngr1*, interferon γ receptor 1; *Ldlr*, low density lipoprotein receptor.

^b^↑↑, Increased disease development; ↓↓, reduced disease development.

^c^The biology of ACAT1 versus ACAT2 differs between mouse and human [110,111].

^d^Note concerning nomenclature: acyl-CoA cholesterol acyltransferases 1/2 (ACAT1/2) are more properly known as sterol *O*-acyltransferases 1/2 (SOAT1/2) and the name ACAT conflicts with the official symbol for a different enzyme (acetyl-CoA acetyltransferase 1/2; ACAT1/2); see http://www.ncbi.nlm.nih.gov/gene/6646 and http://www.ncbi.nlm.nih.gov/gene/8435. However, because the literature largely continues to use ACAT1/2 (rather than SOAT1/2) for acyl-CoA cholesterol acyltransferases 1/2 this usage is followed here.

^e^Data not available.

^f^The ApoE paradox. *APOE4* is a risk factor for both AD and ATH, but knockout promotes ATH but reduces AD in mouse models.

^g^Some data are contradictory; text for details.

*By inference; the production of Aβ (processing product of human APP) is central to AD pathology.

**By inference; *CYP7B1* expression is downregulated in AD patient brain [104].

One may conclude that several common genes act in parallel to predispose to both disorders, but that there is subtle divergence in the molecular pathways leading to one or other disease, notably at the level of APOE. This presents a conundrum that is not yet understood because APOE4 is a risk factor for both diseases.

### Site of action: the immune system

GWAS studies and animal models have confirmed that key genes are involved in both ATH and AD and, in addition to cholesterol metabolism, these also address inflammation and immunity. The evidence demonstrates that the immune system centrally determines disease outcome in both cases.

#### Both diseases have an inflammatory component

Inflammatory pathways have been implicated in both ATH and AD. For example, C-reactive protein (CRP) levels are markedly altered in both diseases. CRP, a marker of inflammation induced by interleukins IL-1 and IL-6, binds to phosphocholine, a component of the bacterial cell wall, and has immunomodulatory properties (reviewed in [112]). In ATH, upregulation of CRP has been known for several decades [113]. For example, CRP immunoreactivity was present in 90% of atheromatous plaques but in only 3% of normal specimens [114]. In AD, there is no evidence for systemic CRP upregulation in blood or CSF, but CRP mRNA levels in brain, particularly in hippocampus, an early site of AD pathology, were increased by over 20-fold versus controls [115], pointing to local inflammation in the brain. For more extensive summary on inflammation in AD and ATH the reader is referred to recent reviews [116-119].

#### Immune downregulation attenuates ATH and AD

Multiple studies confirm the central involvement of the immune system in both diseases and, moreover, that impaired immune function abrogates both diseases. For ATH, M-CSF deficiency resulted in significantly reduced atherogenesis [120]. Song *et al.*[121] crossed Rag1-deficient mice (that lack mature T and B lymphocytes) with *Ldlr*^−/−^ mice, generating animals in which ATH lesion development was markedly reduced; similar findings were reported for Rag-1 deficient *Apoe*^−/−^ mice, although only significantly in males [122]. Mature B cell depletion using a CD20-specific monoclonal antibody induces a significant reduction of ATH in various mouse models of the disease [123]. IFN-γ receptor knockout mice exhibited a substantial reduction in ATH lesion size [105]. Similar findings have been reported for other immune system components (not reviewed).

In AD models there have been some inconsistent findings. For example, deficiency of Ccr2 (chemokine C-C motif receptor 2, a protein expressed principally on microglia) was reported to accelerate disease [124]. However, other findings support the view that immune system downregulation prevents AD development. Knockout of IFN-γ receptor reduced gliosis and amyloid plaques [106], and blockade of TNF-α reduced Aβ-induced cognitive impairments [125]. Ablation of CD14, a key molecule in innate immunity, led to decreased plaque burden [126]. DOCK2 (dedicator of cytokinesis 2) is expressed in brain microglial immune cells and modulates cytokine secretion and phagocytosis; knockout was reported to result in reduced plaque area and size [127].

In both diseases, therefore, inhibition of the immune system generally attenuates disease processes. This argues that activation of the immune system is centrally involved in the pathoetiology of both diseases.

#### Site of action: the immune system determines disease development

The suggestion that the immune system (and potentially infection; below) are implicated in the pathoetiology of both diseases prompts the speculation that key deficiencies, such as of *Apoe* or *Ldlr*, only in immune cells, might alone reiterate the disease phenotype of the animal models.

The techniques are available to address this issue. If a knockout mouse is irradiated, and then transplanted with bone marrow cells from a wild type mouse, the immune system regenerates, producing a mouse in which the immune system alone carries the wild type allele. Conversely, knockout bone can be transplanted into a wild type mouse, producing an animal in which the knockout is only present in bone marrow-derived cells.

Van Eck [128] transplanted *Apoe*^+/+^ bone marrow into atherosclerosis-prone *Apoe* null mice, and observed that bone-marrow transplantation led to a marked reduction in ATH lesions. Herijgers *et al.*[129] transplanted bone marrow from *Ldlr*^−/−^ mice into irradiated wild type mice and, despite no significant changes in serum cholesterol or lipoprotein profiles, animals developed atherosclerotic lesions. A similar finding was reported by Fazio *et al.*[130]. Zhao *et al.* transplanted bone marrow from ABCA1/SR-BI double knockout mice into ATH-prone mice and reported that this increased disease development, despite an unexpected reduction in circulating cholesterol levels [131].

Similar findings using bone-marrow transplantation have been reported in AD models. Keene *et al.*[132] transplanted prostaglandin receptor (PTGER2)-deficient (*EP2*^*−/−*^) bone marrow into irradiated *APP*^*AD*^ mice, and observed that lesion sizes were substantially reduced in mice receiving knockout bone marrow compared to mice receiving *EP2*^+/+^ marrow. Hao *et al.*[133] performed a similar experiment with bone marrow deficient in myeloid differentiation factor 88, and reported that the deficiency (in bone marrow-derived cells alone) markedly reduced amyloid burden. Wild type bone-marrow transplantation into *APP*^AD^ mice markedly reduced cerebral pathology and, conversely, mutant (PS1) bone marrow exacerbated disease [134]. In the most recent study, *APP*^AD^ mice received bone-marrow transplants from mice expressing either human APOE4 or APOE3. Transplantation markedly reduced pathology, but the APOE3 transplants were far more effective [135].

Therefore, for both diseases, at least in mouse models, the genotype of bone marrow-derived cells determines disease development, and not that of the host. This is despite the fact that transplanted knockout animals generally maintain the marked changes in levels of blood cholesterols and lipoproteins characteristic of the host knockout mouse, demonstrating that these systemic changes are not directly responsible for disease development.

#### Central role of macrophages

Macrophage infiltration and foam cell formation are known to play a central role in ATH disease development (for recent comprehensive review see Moore and Tabas [136]). The situation in AD is more contentious, but the available evidence indicates that, here again, macrophages play the central role.

Macrophage infiltration is a feature of AD brain. Macrophage numbers are dramatically increased in AD brain [137], as seen in HIV-1 encephalitis; infiltration is most abundant in perivascular regions and locates to endothelial tight junctions, Aβ plaques, and macrophages that partially encircle the walls of Aβ-rich CAA [137]. Zaghi *et al.*[77] demonstrated that, in human AD brain, macrophages strongly home to deposits within and surrounding the brain vasculature where they colocalize with Aβ (Figure 4B).

Studies in mouse genetic model confirm a central role for macrophages in both diseases. In ATH, a human *APOE* transgene under the control of the macrophage lysozyme promoter was crossed into *Ldlr*^−/−^ mice; this significantly reduced ATH lesion area [138]. The same finding was reported with macrophage-specific *Apoe* gene repair in APOE-knockdown mice [139]. Knockouts of PPARγ or LRP1 only in macrophages increased lesion size in ATH-prone mice [140,141].

In AD the situation is complicated because the brain contains both resident brain-specific macrophage-like cells, the microglia, and true macrophages that infiltrate from the circulation. Wegiel *et al.* have argued that microglia actively promote disease development in *APP*^*AD*^ mice and play a pivotal role in amyloid deposition [142,143]. Simard *et al.*[144] argued instead that bone marrow-derived microglial cells are protective and can remove amyloid deposits. However, Grathwohl *et al.* used a microglia-specific cell ablation technique in *APP*^AD^ mice; nearly complete ablation of microglia had no effect on AD disease development [145]. Hawkes and McLaurin [146] argued that infiltrating peripheral macrophages, rather than microglia, play a central role in clearing Aβ deposits.

In a pivotal study, Town *et al.* used macrophage-specific expression of a dominant-negative form of TGF-β in *APP*^AD^ mice; expression in microglia was absent. Ablation of macrophage TGF-β signaling markedly inhibited disease development [147], confirming that macrophages alone can play a determinant role in AD disease development. For recent reviews on macrophage recruitment into AD brain see [148,149].

The central involvement of the immune system (and specifically of macrophages) unavoidably prompts the question of whether some infectious component might contribute to disease development in ATH and/or AD. The idea that a common disease condition might have an infectious component is not new. Although initially challenged [150], Warren and Marshall in 1984 observed that biopsy specimens of patients with gastric ulcers contained spiral or curved bacteria [151], and received the Nobel prize for their discovery that *Helicobacter pylori* is a cause of gastric ulcers.

### Transmissibility of AD

AD has features of transmissibility. There is intriguing evidence that pathology spreads progressively through the brain from initial foci. Duff and colleagues [152] report that Tau pathology in human AD brain commences in the entorhinal cortex and spreads trans-synaptically from cell to cell. Similar findings were reported in mice by de Calignon *et al.*[153] who expressed a mutant form of human Tau that predisposes to AD-like pathology in the entorhinal cortex. Pathology propagated from transgene-expressing neurons to adjacent brain regions lacking any detectable transgene expression.

There is direct evidence of transmissibility. Marmosets do not normally develop AD pathology but, when injected intracerebrally with brain tissue from a patient with early-onset AD, animals developed AD-like amyloid plaques (but no NFT) 6–7 years after inoculation [154]. Non-AD brain tissue failed to transmit disease and the induced degeneration was transmissible to further animals [155].

Further evidence for transmissibility emerges from APP transgenic models in which mice develop AD-like pathology only late in life ([156] for review). Disease onset was remarkably accelerated by inoculation of extracts of human AD brain into young *APP*^AD^ transgenic mice [157,158]. Using 10% w/v brain homogenates from postmortem AD patients, Meyer-Luehmann *et al.*[158] demonstrated that inoculation into young transgenic mice induced robust deposition of Aβ, whereas non-AD brain failed to do so. Similar seeding has been reported when brain extracts from older (diseased) APP transgenic mice are injected into young transgenic animals [158-161]. Parallel observations have been reported in transgenic mice and rats that do not alone develop disease [162,163]. Although classic AD-like Tau pathology is generally absent, marking differences between human and murine Tau, NFT were induced when extracts of transgenic mouse AD brain were inoculated into transgenic *APP*^*AD*^ mice expressing mutant Tau [164].

Although pretreatment of brain extracts with antibody to Aβ could inhibit seeding, confirming a role for Aβ, experiments using synthetic Aβ peptides (1–40 or 1–42), in either soluble or aggregated forms, failed to transmit infection; neither did oligomeric forms of Aβ prepared from cell cultures overexpressing APP [158]. Failure of synthetic Aβ to transmit disease has been confirmed [155]. Rosen *et al.*[163] state: ‘At present, there is no evidence that AD *per se* is transmissible in the same manner as is prion disease’, and others have suggested that a second factor is likely to be required [165].

These experiments need to be interpreted with caution because Aβ deposition is not an accurate proxy for Alzheimer-type dementia; some individuals with extensive amyloid deposits fail to show significant cognitive impairment (reviewed in [166]) and clinical trials to remove amyloid deposits have failed to lead to disease improvement. Equally, in the animal models discussed above there has been no demonstration that the animals suffer from a condition that strictly reproduces human dementia. Despite this caveat, the transmissibility of AD pathology has been widely replicated and, at face value, given the failure of Aβ peptides (either soluble or aggregated) to transmit disease, might suggest that a second agent (possibly infectious) may be required for full transmission.

### Evidence for an infectious component to AD

For AD, the idea that microorganisms might participate in senile dementia was first proposed by Fischer in 1907 [167]. More than a century later, a volume of data supporting this hypothesis has begun to accumulate [168-170], and reports have appeared of associations between AD and diverse infectious agents including both viruses and bacteria [171]. Evidence for a causal link between infection and disease is generally based on two types of observations. (i) Statistical association between an infectious agent and clinical disease; however, such associations could be fortuitous, and are often regarded as unconvincing. (ii) A second type of study – intervention – is necessary to demonstrate causation. We therefore address both types of evidence. Although not fully comprehensive, the selection aims to highlight both the diversity (and the inconsistencies) in the literature.

#### Herpes simplex virus type 1 (HSV-1)

Latent herpesvirus HSV-1 is widespread in the population and virus reactivation is associated with lesions of the skin and the central nervous system (CNS). Mann *et al.*[172] and Esiri [173] provided the first evidence of HSV-1-immunopositive neurons in AD brain. HSV-1 DNA was detected in brain tissue of 3/3 patients with familial AD but all but one of six age-matched controls were negative [174]. Other studies have suggested that the presence of HSV-1 DNA in AD and control brain samples is unrelated to disease status (e.g. [175]). A complication is that a majority of the population is HSV-1 seropositive. In an alternative approach, Letenneur *et al.* used IgM seropositivity as a marker of recent herpesvirus activation in a large cohort of healthy elderly. Those who were IgM-positive were significantly more likely to develop AD during the follow-up period of 14 years (relative risk 2.55; 95% CI 1.38–4.72) [176].

#### Chlamydophila (Chlamydia)

Until recently known as a *Chlamydia* species, *Chlamodophila pneumoniae* is an obligate intracellular bacterium associated with respiratory infections of humans and animals. Using PCR and electron microscopy, Balin *et al.* identified *C. pneumonia* in 90% of postmortem AD brain samples but in only 5% of control samples [177]. Both typical intracellular and atypical extracellular forms were found in astrocytes, perivascular macrophages, microglia, and neurons; moreover, cells carrying the bacteria colocalized with amyloid plaques and NTFs [178,179].

#### Spirochetes

**Figure 5:**
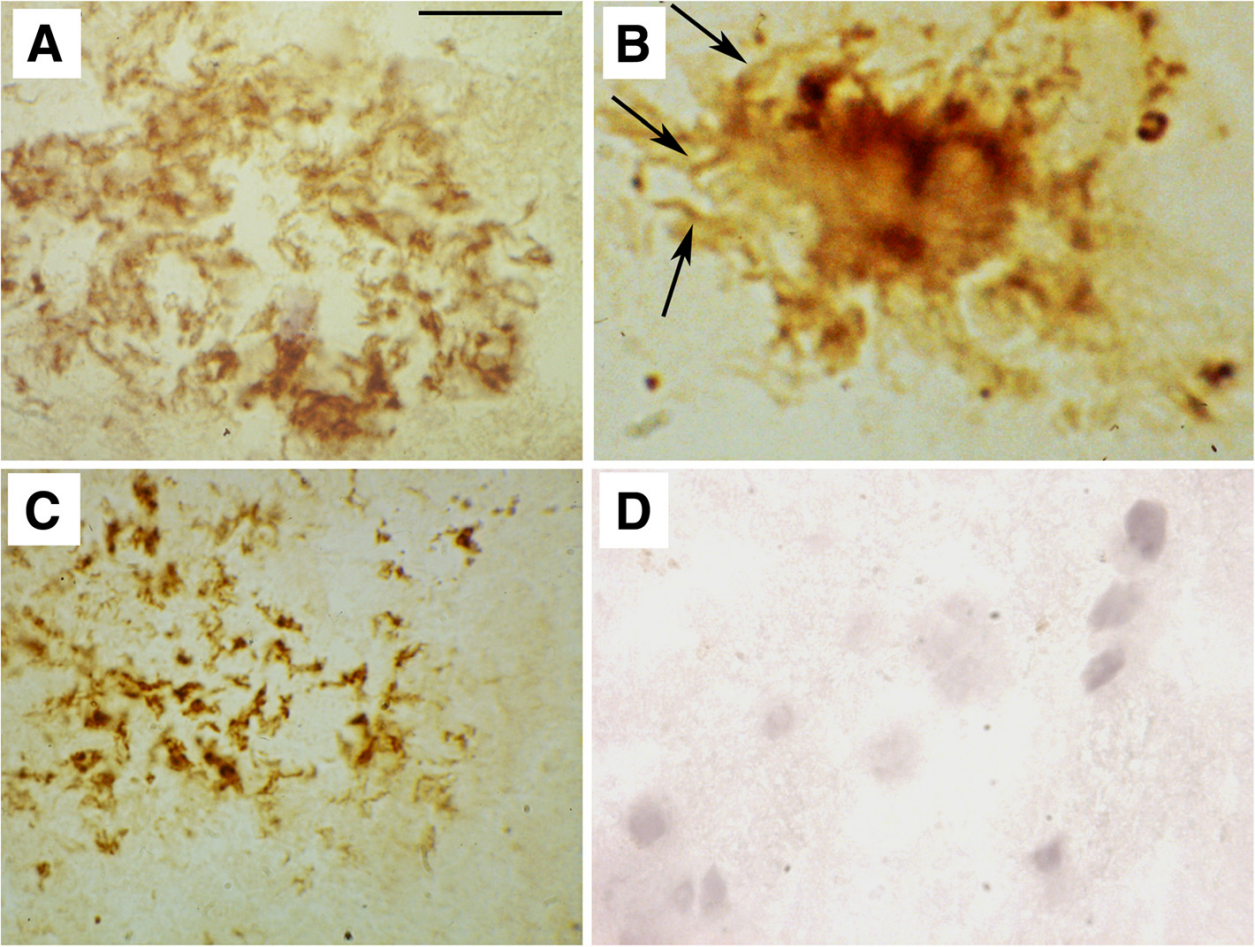
**Spirochetes in Alzheimer disease brain. (A)** Detection in an immature senile plaque using a cocktail of specific antibodies against *Borrelia burgdori* (dark-brown staining). **(B)***Borrelia* sequences in a mature plaque detected using *in situ* hybridization using a *B. burgdorfi* probe. **(C)** Detection in an amorphous plaque using an antibody to bacterial peptidoglycan. **(D)** Control brain stained with antibodies against *B. Burgdorfi.* Arrows in B indicate bodies resembling helical spirochetes. Scale bar, 80 μm for **(A, D)**, 30 μm for **(B)**, and 20 μm for **(C)**. Figure kindly provided by Judith Miklossy, Switzerland.

This diverse group of double-membrane spiral-shaped bacteria are generally free-living, but are responsible for several important diseases including Lyme disease (principally *Borrellia burgdorferi*) and syphilis (*Treponema pallidum*). Rivière *et al.* reported that several species of oral *Treponema* were present in brain samples from both AD (14/17 positive) and controls (4/18), but the quantity of bacterial DNA was significantly higher in AD patients [180]. In another study, using a panel of methods, spirochetes were detected in CSF, blood, and brain of 14 AD patients, whereas 13 control samples were all negative [181]. In 3 of 14 samples the bacteria were identified as *Borrelia burgdoferi*. Spirochetes colocalized with senile plaques and NTFs and were present in vascular walls in association with amyloid deposition [168] (Figure 5).

#### Helicobacter pylori

Also with a helical or spiral structure, the Gram-negative Spirilla *Helicobacter pylori* is a causal agent for gastric ulceration and has also been highlighted as a potential risk factor for AD development. It was reported that 88% of AD patients were positive by histology for *H. pylori* versus 47% of controls [182]. In a group of 53 AD patients, *H. pylori* infection was significantly associated with reduced cognitive ability and higher CSF Tau [183] and, in the most recent study, in which 600 elderly individuals were followed for a period of 19 years, *H. pylori* infection determined by serology was found to be a risk factor (risk ratio 1.46) for developing dementia [184]. However, another study failed to find any association between *H. pylori* infection and AD [185].

#### Intervention

Infection can precipitate AD-like pathology in animal models (see later), but few studies have addressed possible intervention in the clinic. Kountouras *et al.*[186] reported that eradication of *H. pylori* infection was associated with a significant reduction in mortality risk in 46 patients with probable AD (risk ratio, 0.287; 95% CI 0.114–0.725).

### Does ATH also have an infectious component?

For over 100 years there have been reports of an association between acute infectious disease, atherosclerosis, and stroke. To our knowledge, direct transmission from primary disease material has not been attempted in ATH models, and (in contrast to AD) there is so far no evidence that ATH can be ‘seeded’ by inoculation of extracts of diseased arteries; we feel such studies may need to be carried out. However, investigations have implicated diverse infectious agents in the pathoetiology of ATH.

#### Herpesviruses

**Figure 6:**
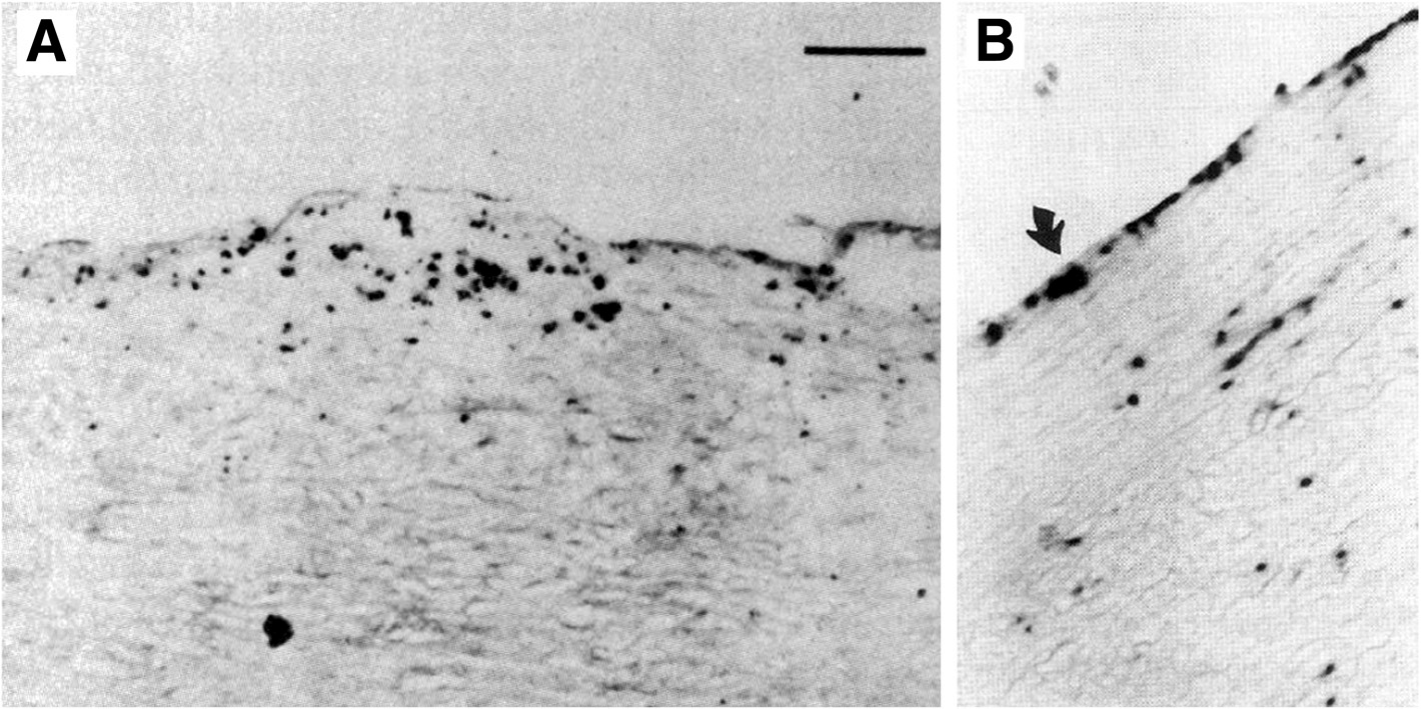
**Virus detection in ATH lesions. (A)** Herpes simplex virus sequences in a thoracic artery lesion from a patient undergoing coronary bypass surgery detected by *in situ* hybridization. Scale bar, 25 μm. Panel adapted, with permission, from [187]. **(B)** Cytomegalovirus sequences in arterial wall from a patient with severe atherosclerosis detected by *in situ* hybridization. Original magnification 100×. Panel adapted, with permission, from [188].

Benditt *et al.*[187] detected herpes simplex virus (HSV) by *in situ* hybridization of aortic samples (Figure 6), but failed to detect DNA sequences of another herpesvirus, cytomegalovirus (CMV); two of four samples with abnormally thick intima media were strongly positive for HSV. Melnick and colleagues [189,190] reported antigens and CMV sequences in association with ATH, and Speir *et al.*[191] showed that one third of atherosclerotic lesions obtained by coronary atherectomy contained CMV DNA sequences. CMV infection was identified as an independent risk factor for restenosis after coronary angiopathy [192] and CMV-positivity is associated with endothelial dysfunction and an increased atherosclerotic burden [193]. Nieto *et al.*[194] found a graded and significant relation between the odds of intima media thickening and the level of CMV antibodies. However, Hendrix *et al.*[188] reported comparable detection frequencies (ca 30–50%) of CMV sequences in arterial samples from both patients with atherosclerosis and non-ATH controls. Because a majority of the population is already positive for CMV and/or HSV subtypes, it is difficult to ascertain whether herpesviruses are bystanders or might potentially be causally implicated in ATH pathology.

#### Chlamydophila

Using electron microscopy, Shor *et al.*[195] first reported the presence of chlamydia-like structures in seven samples of fatty streaks and atheromatous plaques that were confirmed by immunocytochemistry in five cases. The same group [196] reported *C. pneumoniae* antigen and sequences in 35–50% of lesions, as confirmed in several follow-up studies [197]. For example, PCR detected *C. pneumoniae* DNA in 31% of atherosclerotic plaques but in only 2% of normal aortic samples [198]. *Chlamydophila* is known to be able to infect and replicate within monocyte/macrophages, endothelial cells, and vascular smooth muscle cells (SMC) [199]. Review of all published studies [200] recorded that, overall, 46% of plaques were positive whereas <1% of control arteries were positive. Recent review of the field [201] emphasized great variation between studies, but that there has been a ‘high degree of consistency in the association between *C. pneumoniae* and arterial atheromatous lesions’.

#### Spirilla and Spirochetes

There have been intermittent reports of an association between ATH and Spirilla and Spirochetes (*H. pylori* and the dental pathogen *Treponema denticola,* respectively). For example, Ameriso *et al.*[202] reported *H. pylori* DNA in 20/38 atherosclerotic plaques whereas 0/7 normal arterial samples were positive. However, another study failed to detect *H. pylori* in ATH plaque, although *C. pneumonia* was found in 50% [203]. Okuda *et al.*[204] studied *T. denticola* sequences and reported that 23% of atherosclerotic lesions were positive by PCR whereas 0/14 control aorta samples were positive.

#### Porphyromonas gingivalis

*P. gingivalis*, a Gram-negative anaerobe implicated in peridontal disease, has also been proposed to be involved in other diseases including ATH, diabetes, and rheumatoid arthritis [205]. An association between dental health and cardiovascular disease was first established at the end of the 1980s [206,207]. Indeed, periodontal pathogens including *P. gingivalis* have been detected in different cardiovascular disease cases including atherosclerotic lesions, aneurysms, and endocarditis [208-210]. As reviewed [211], *P. gingivalis* has been associated with SMC proliferation and endothelial cell apoptosis. The large INVEST study reported a direct relationship between carotid intima thickness and peridontal bacterial burden [212].

#### Intervention studies

Given the potential role of bacterial infection in ATH, there have been several trials of antibiotic treatment, but without significant benefit [201,213,214]. However, key bacteria (e.g*. C. pneumoniae*) can persist for long periods as a latent intracellular infection, and it is unclear what degree of clearance was achieved in these studies. The best available data are from chicken (and from mouse models, below). Infection of chickens with Marek disease virus, a herpesvirus, causes them to develop atherosclerotic lesions that resemble ATH in human [215] (reviewed in [216]).

### Infectious agents contribute to AD and ATH

Wild type mice inoculated with *C. pneumoniae* cultivated from AD brain developed amyloid plaques [217]. HSV-1 infection of cultured neuronal and glial cells leads to a dramatic increase in the intracellular levels of Aβ, and antiviral therapy blocked Aβ production [218]. Infection with neuroadapted mouse hepatitis virus strain JHM was also reported to exacerbate AD-like pathology in a transgenic mouse AD model [219]. Of note, Aβ deposition is a common feature of brain infection with HIV in human [220]. Conversely, immunosuppressive *Toxoplasma gondii* inhibited disease development in an *APP*^AD^ mouse model [221].

For ATH, diverse experiments in animal models have demonstrated that inoculated infectious agents such as *C. pneumoniae* persist in atherosclerotic lesions [222] and accelerate ATH development in susceptible mice. For example, *C. pneumoniae* infection increased aortic ATH in the *Ldlr*^−/−^ mouse model [223,224], although this has been disputed (e.g., [225,226]), and infection can stimulate cholesterol-rich foam cell formation [227] and SMC proliferation [228], markers of ATH. Infection of ATH mouse models with *Porphyromonas gingivalis*[229,230], *H. pylori*[231], or *Streptococcus mutans*[232] also accelerated atherogenesis. Similar results have been obtained with viral pathogens. Virus infection of ATH-prone mice promotes atherogenesis, exemplified by mouse gammaherpesvirus-68 [233], influenza virus [234], and CMV [235,236].

Conversely, as with AD, infection with an immunosuppressive pathogen (here *Schistosoma mansoni*) reduced ATH lesions by 50% in infected mice [237].

#### Non-specific immune activation predisposes to disease

Infection is not strictly required for atherogenesis. Wright *et al.*[238] reported that the profile of ATH development was unaffected in *Apoe*^−/−^ mice additionally carrying the *lps*^*d*^ mutation that renders them unable to respond to bacterial lipopolysaccharide (LPS) – although LPS is only one of multiple stimulators of innate immunity. Germfree *Apoe*^−/−^ mice, that are held to be free of bacteria, viruses, and fungi, developed atherosclerosis [238]. However, non-specific immune challenge can precipitate disease. LPS injections alone can increase atherosclerotic lesion size [239]. Vliegen *et al.*[240] compared the effects of inoculation of mouse CMV (MCMV) with ultraviolet light (UV)-treated MCMV. The inactivated virus increased atherosclerotic lesion area and T cell number in the atherosclerotic lesions, whereas only live MCMV infection increased T cell numbers in the internal organs.

Similar findings have been made in AD models. Transmission of disease (see earlier) has been reported but, importantly, heating AD brain extracts to 95°C reduced (45%) but did not eliminate transmission [158], arguing for a non-specific inflammatory effect. In support, systemic immune stimulation with the viral mimetic, poly(I:C), during gestation predisposes to AD-like neuropathology [241]. Prenatally stimulated animals had increased levels of Aβ, hyperphosphorylated Tau, and NFT formation. Lee *et al.* showed that injection (i.p.) of LPS into mouse models led to increased levels of Aβ and Tau aggregation [242].

In conclusion, in both ATH and AD, there is strong evidence linking disease development to infection, and overwhelming indications that infectious agents home to diseased tissue and aggravate pathology. Nonetheless, one suspects that any one of several agents can accelerate atheroma formation – and local immune cell activation (via either infectious or non-infectious agents) precipitates disease. In short, infectious agents *per se* may not be required for disease development (with the caveat that several agents are intrinsic to the mammalian genome, e.g., endogenous retroviruses, not reviewed) but, in the absence of other risk factors, transmissible agents are more than likely to play a determining role – as stated by Epstein *et al.* ‘compelling data indicate that infection does contribute to atherogenesis and to the acute complications of atherosclerosis caused by plaque rupture’ [243].

#### Focal nature of disease

Both AD and ATH are manifested focally, and this affords a further argument. In both conditions, numerous foci of disease replicate the same pattern of progression at different locations. At the same time, there are significant stretches of tissue which are not affected by the disease, despite the presence of all confounding factors for decades since the beginning of the pathological process. The focal nature excludes somatic mutations or other cell-autonomous defects in the cells forming a solid tissue. Instead, a stochastic element, particularly at the initial stage of the disease, is most plausible, and foci of infection are an obvious contender. Subsequent stages may not require direct pathogen involvement, because local inflammation, once established, may persist via the involvement of activated immune cells.

### Drug overlap

If the two disorders have a similar etiology, drugs (other than antibiotics, discussed above) effective in one disorder might be expected to show efficacy in the other. Both diseases are associated with elevated blood cholesterols (even though transplant experiments in mice have demonstrated that this is not causal, above), raising the question of whether blockade of cholesterol synthesis might be used to treat ATH or AD.

Statins reduce body excess of cholesterol by inhibiting a key enzyme in *de novo* cholesterol synthesis, HMG CoA reductase. No conclusive benefits have been reported in AD [244] whereas, in ATH, some benefits have been reported, notably in the ASTEROID trial of rosuvastatin [245,246], although other trials failed to give unequivocal results [247]. Statins have many side-effects and do not appear to be the panacea one might have hoped for. This makes sense given that, in genetic models of both diseases, transplantation of wild type bone marrow abrogates pathology despite the persistence of host hyperlipidemia.

#### Curcumin

An aromatic component of the spice turmeric (*Curcuma longa*), this molecule has been suggested to prevent AD Aβ toxicity. Curcumin can reduce amyloid *in vivo* in transgenic AD models and remove existing plaques [248-251]. Curcumin also reduces Aβ-mediated blockade of long-term potentiation (LTP) [252], a likely electrophysiological correlate of learning and memory. Trials of curcumin in AD patients have been explored [253] and further studies are ongoing.

Curcumin also exerts protective pharmacologic effects against ATH. In different mouse models, curcumin can potently inhibit ATH disease development [254-256]. Although several possible targets have been discussed, including inhibition of NF-κB (a marker and mediator of innate immunity induction) [257] the precise molecular target(s) for the beneficial effects of curcumin remains unknown.

#### Resveratrol

Resveratrol is a diphenolic molecule and notably a component of red wine. Intriguingly, resveratrol promotes Aβ clearance in cell culture [258] and protects against Aβ toxicity in culture [259,260] and in adult rats [261]. Similar findings have been reported in transgenic mouse AD models treated with resveratrol [262] or even, perhaps controversially, Cabernet Sauvignon [263]. The molecule is in clinical trials in AD [264].

For ATH, the potential protective activity of resveratrol has been discussed for three decades. Like curcumin, resveratrol has been shown to reduce atheroma formation in different mouse models of atherosclerosis (*Apoe*^−/−^*Lldl*^−/−^ on a high-fat diet) [265-267], in some cases dramatically [268]. Protective effects in hypercholesterolemic rabbits have also been recorded [269], and several clinical trials are ongoing in diverse indications. The specific molecular target is not known but, among other activities, resveratrol has been reported to inhibit ACAT [270].

#### Acyl-CoA cholesterol acyltransferase (ACAT) inhibitors

ACAT (also known as SOAT, see footnote on nomenclature in Table 1) is a key enzyme catalyzing the esterification of cholesterols. In mouse models, inhibition of the enzyme (ACAT1/2) attenuates both ATH and AD. For ATH, to give only two recent examples, in *Apoe*^−/−^ mice the inhibitor F1394 retarded ATH plaque progression [271]; similar observations were made with the inhibitor Manzamine A [272]. Knockout studies for ACAT1 and ACAT2 have generally revealed a protective role of gene disruption (although the literature is discordant for ACAT1; Table 1). In AD, the ACAT inhibitor CI-1011 [273] modulates Aβ production [274] and reduces Aβ accumulation in a transgenic model of AD [275,276]. Similar anti-Aβ effects were observed with a second ACAT inhibitor, CP-113,818 [275]. It was recently reported that knockdown of ACAT1 expression *in vivo* using a viral vector alleviated AD-like pathology in a mouse model [277], confirming that ACAT1 and ACAT2 are both likely drug targets in AD and ATH.

#### Acetylcholinesterase (AChE) inhibitors

Given well-established deficits in central cholinergic neurotransmission in AD, AChE inhibitors such as donepezil, galantamine, and rivastigmine have been widely trialed in AD – with evidence of efficacy in slowing disease progression (reviewed in [278]). In ATH, perhaps surprisingly, donepezil infusion could attenuate atherogenesis in susceptible mice [279].

The mechanism may not be what we think. Interestingly, the target enzyme AChE reiterates the structure of the catalytic site of a steroid gating enzyme (HSD11B), and molecular design directed to the AChE site yielded HSD11B inhibitors (three for HSD11B1 and four for HSD11B2) [280]. Intriguingly, polymorphisms in the gene encoding the ‘backup’ acetyl choline hydrolyzing enzyme butyrylcholinesterase (*BCHE*) are reported as risk factors in both ATH [34,281] and AD [282]. Cholesterol hemisuccinate is a weak inhibitor of BChE (IC50 168 μm) but a potent inhibitor of AChE (IC50 0.79 μM) [283]. AChE inhibitors may therefore act, in part, via interference with steroid and sterol metabolism.

AChE is widely expressed at the surface of platelets and red blood cells, macrophages express specific nicotinic acetyl choline receptors, and systemic cholinergic signaling modulates platelet aggregation, macrophage function, and innate immunity (see [284]). Interaction with these pathways, either directly or via CNS effects, could underlie the beneficial effects of AChE inhibition in both ATH and AD.

The overlaps in drug responsiveness between AD and ATH reinforce Roher’s earlier observation that there is ‘an immediate need for prospective clinical trials to assess the efficacy of AD prevention using antiatherosclerotic agents’ [285]. Equally – do other anti-AD drugs combat ATH?

### Transcriptome module overlap

Further evidence of commonality between AD and ATH is provided by gene expression analysis. Ray *et al.*[286] used a systems biology approach to analyze brain RNAs from 20 confirmed AD brains versus 13 controls. 1600 genes differentially expressed in AD were identified and classified according to functional module. The two predominant modules, confirmed by functional annotation clustering, were (i) AD/neurodegeneration, as expected, but also (ii) cardiovascular/coronary artery disease [286]. The authors concluded that many pathways are common to both diseases; their results provide strong support for a mechanistic linkage between AD and ATH.

### Mechanisms – inflammation, cholesterol metabolism, immunosterols

Both diseases are underpinned by genes affecting cholesterol transport/metabolism and immunity. Immunostimulation precipitated by infectious agents or specific components such as LPS can increase, sometimes dramatically, the development of ATH or AD in animal models. Equally compelling are the data that the immune system, notably macrophages, is centrally involved in the disease processes that culminate in local inflammation, the formation of cholesterol-loaded foam cells, and vascular occlusion. These observations point to a direct link between infection and cholesterol metabolism, as borne out by studies on APOE.

#### APOE and infection

*APOE* alleles, encoding a key lipid transport molecule, play a crucial determining role in the outcome of viral and bacterial infection. In mouse models, APOE modulates infection by HSV-1 [287], *Chlamydophila*[288], *Klebsiella pneumoniae*[289], *Listeria monocytogenes*[290] and *Leishmania*[291]. In *Apoe*^−/−^ transgenic mice expressing human APOE, APOE3/APOE4 genotype has a marked influence on HSV-1 propagation and latency – APOE4-expressing mice challenged with HSV-1 had very high levels of virus in brain compared to APOE3-expressing or knockout mice [287,292]. Effects of LDLR mutation on *Toxoplasma* disease were also reported [293].

Similar findings have been made in human. The APOE4 allele is reported to accelerate HIV proliferation whereas, by comparison, APOE3 is protective [294]. Numbers of *Chlamydophila*-infected cells and bacterial load were significantly higher in homozygous *APOE4* patients than in *APOE2* or *APOE3* carriers [295]. Conversely, for malaria, *APOE3/4* afford protection and *APOE2* homozygotes are most susceptible [296].

Thus, for some pathogens (e.g., HIV, HSV-1, *Chlamydophila*) *APOE4* predisposes to disease, whereas for others (e.g., malaria) *APOE4* is protective. Understanding how *APOE* allelic variants can differentially confer susceptibility to one disease, but resistance to another, is clearly of fundamental importance. This raises a central question – how do APOE and cholesterol metabolism relate, at a molecular level, to infection and immunity?

#### Cholesterol signaling

Adequate cholesterol supply can play an important role in the assembly of the membrane components required by many (but not all) pathogens; some infectious agents are known to exploit transport and uptake mechanisms such as those mediated by APOE and LDLR. However, evidence is now emerging that cholesterol and its oxysterol derivatives play potent roles as specific signaling molecules in the immune system.

Cholesterol itself is poorly soluble, but is prone to spontaneous and enzyme-mediated oxidation at the 7, 11, 5–6, 22/24/25/27 positions [297] that increase mobility: for example, efflux of oxidized cholesterol from macrophages is ~50× faster than of cholesterol itself [298]. Side-chain oxidation is also required for export from the brain.

Oxidized cholesterols are potent signaling molecules. One major pathway is via the liver X receptors (LXR). Although prominently expressed in the liver, where LXRα and LXRβ regulate bile acid synthesis and metabolism/excretion (reviewed in [299]), in peripheral tissues receptor activation feeds back to repress cholesterol synthesis and promotes export of excess cholesterol to the liver and bile for excretion. The best natural LXR ligands are cholesterols oxidized at the 22, 24 and/or 25 positions [300], although 27-hydroxycholesterol (27OHC) has been argued to be the endogenous ligand [301,302].

#### Specific role of ‘immunosterol’ 25OHC in innate immunity

Elevated levels of oxysterols are reported in ATH lesions, notably 27OHC and 7αOHC [303], and could contribute to atherogenesis (reviewed in [304]); anomalies found in AD brain include oxysterols, cholesterol precursors, and steroids (e.g., [305-308]. However, new evidence from Ghazal’s group [309] has emerged that one molecule, 25OHC, plays a specific role in regulating responses to pathogens.

Several enzymes are known to catalyze the formation of 25OHC, notably the promiscuous enzyme CYP3A [310], CYP27 that catalyzes 24, 25, and 27 hydroxylation [311], and CYP46 that predominantly catalyzes 24S-hydroxylation but also 25-hydroxylation [312]. By contrast, in macrophages (centrally implicated in both ATH and AD), 25OHC synthesis appears to be principally mediated by the key enzyme cholesterol 25-hydroxylase (CH25H).

CH25H is a most unusual enzyme. Unlike classic heme-dependent P450 enzymes (CYPs), that widely catalyze the hydroxylation of hydrophobic compounds including sterols and steroids, CH25H, located in the endoplasmic reticulum, is a di-iron enzyme [313] whose ancestry goes back to yeast. Whereas many CYP enzymes are promiscuous in substrate specificity and site of modification, CH25H appears to be specific for 25-hydroxylation of cholesterol. We have here a glimpse of intriguing evolutionary constraints that deserve to be followed up.

**Figure 7:**
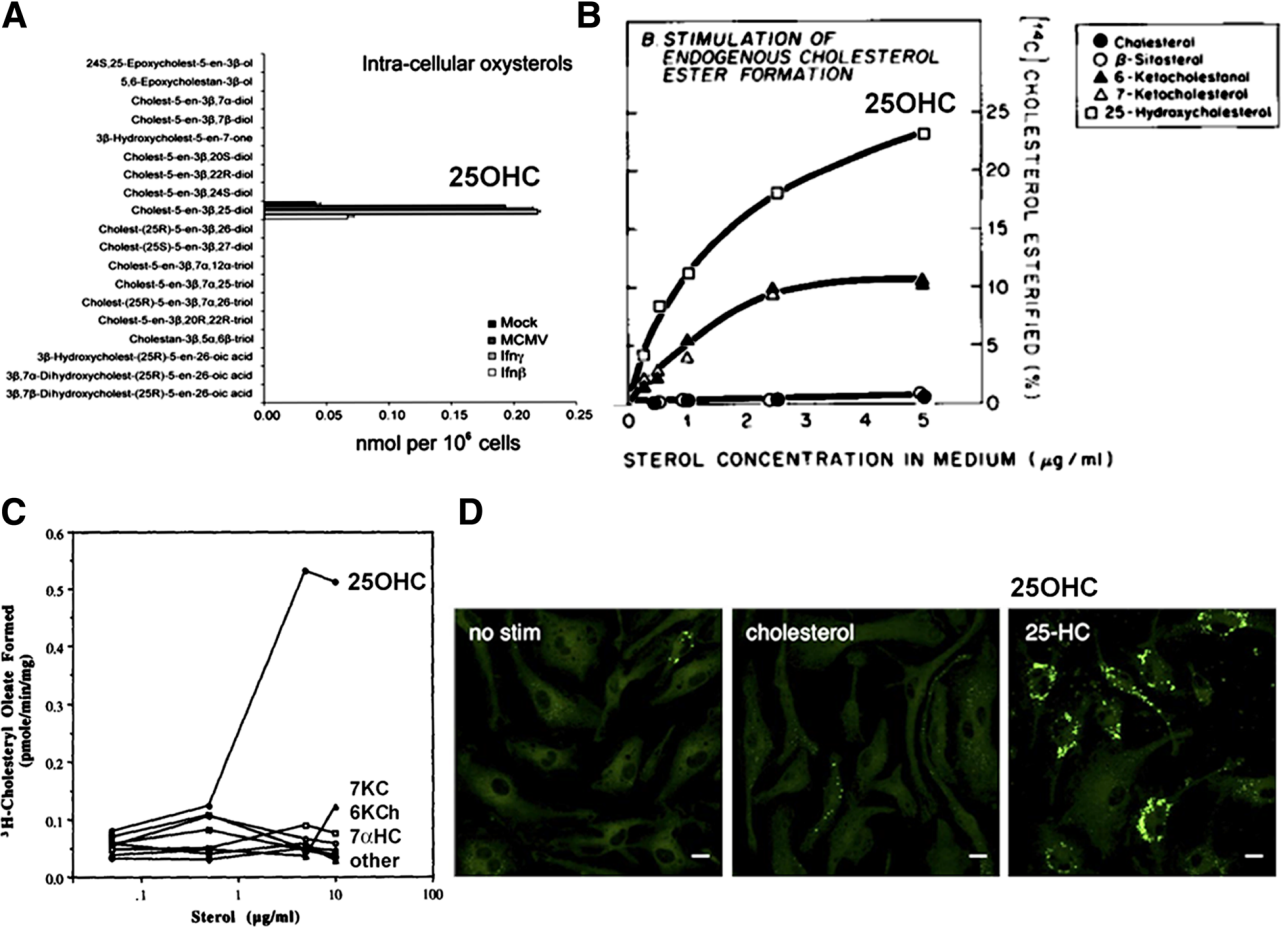
**25OHC induction by immunostimulation or infection promotes cholesterol esterification and foam cell formation. (A)** 25OHC is the only oxysterol induced by interferon (IFN) treatment or infection; panel from [309], with permission. **(B)** 25OHC stimulates cholesterol esterification in intact cells, from [316], with permission. **(C)** 25OHC is an allosteric effector of ACAT activity in insect cells expressing human ACAT, from [317] with permission; 7KC, 7-ketocholesterol; 6KCh, 6-ketocholestanol; 7αHC, 7α-hydroxycholesterol. **(D)** Treatment of bone marrow-derived macrophages with low-dose (ca. 0.1 μM) 25OHC, but not cholesterol itself, induces lipid body formation (BODIPY 493/503 fluorescence); scale bar 10 μm. From [318], with permission.

Macrophage CH25H expression is specifically upregulated by inducers of innate immunity, including LPS, poly (I:C), lipoteichoic acid, specific Toll-like receptor (TLR) agonists, and by IFN-α or IFN-β [257,314,315] (Figure 7A). In mice, intraperitoneal administration of a TLR agonist led to marked upregulation of *CH25H* mRNA, most strikingly in liver (200×), brain (25×), and heart (50×), with a fivefold increase in serum 25OHC levels [257]. Brain expression of CH25H was induced by i.p. LPS injection into mouse [319], and intravenous injection of LPS in healthy human volunteers resulted in 2–3-fold increase in plasma 25OHC [314].

The specificity of the induction is striking. Ghazal’s group [309] screened IFN (β or γ)-treated macrophages by liquid chromatography and mass spectrometry (LC-MS) for all intracellular and secreted oxysterols, revealing 25OHC as the sole oxysterol produced in response to stimulation (Figure 7A). Similar CH25H upregulation and 25OHC synthesis in macrophages was observed in response to viral infection (mouse CMV) [309]. This identifies 25OHC as a specific signaling molecule in response to stimulation of innate immunity.

In this context it is intriguing to note that curcumin, a drug with potential activity against both AD and ATH (see earlier), has been shown able to abolish macrophage expression of CH25H following induction of innate immunity [257]; this could underlie its beneficial affects.

Upregulation of CH25H enzyme plays a protective role. Blanc *et al.*[309] demonstrated that 25OHC supplementation in the sub-micromolar range (generally 0.1–1 μM) induces a broad-specificity antiviral response, inhibiting infections by influenza virus, HSV-1, varicella zoster, and murine γ herpesvirus 68. Similar potent antiviral effects were demonstrated by Cheng’s group for vesicular stomatitis virus (VSV), HIV, and a range of acutely pathogenic viruses [320]. Inhibition of mouse CMV proliferation by 25OHC has been confirmed [321]. Although both 27OHC and 24(S)25-epoxycholesterol do display significant antiviral effects [309], these molecules were entirely absent from stimulated macrophages in the screen of Blanc *et al.*

In further investigation of the mechanism, 25OHC was found to interfere with virus proliferation, and virus plaque size in cell culture was diminished in the presence of 25OHC [309]. It was reported that 25OHC inhibits membrane fusion between virus and cell [320] and, importantly, 25OHC had no effect on the non-enveloped viruses adenovirus 5 or 19a [309]. These findings argue that induction of CH25H in response to infection {or by LPS, poly(I:C), or IFN} protects against viral infection by blocking specific membrane steps in virus entry or maturation. Effects on bacterial pathogens have not been tested, but induction in response to LPS suggests that 25OHC could potentially have antibacterial effects.

#### What is the target for 25OHC?

This work raises the issue of whether there is a specific receptor for 25OHC, or whether 25OHC non-specifically interferes with membrane assembly and fusion processes important for pathogen infection and replication. Blanc *et al.*[309] suggest that 25OHC at higher concentrations (>1 μm) may have non-specific effects. However, at submicromolar concentrations they report that, unlike 25OHC, a structural enantiomer, ‘ent-25HC’, was inactive (although some activity was observed at high concentration) in an antiviral assay, and concluded that the antiviral effects of low-concentration 25OHC are mediated by specific receptor binding.

How does 25OHC mediate antiviral effects? An effect via LXR and cholesterol metabolism *per se* seems unlikely because mice lacking CH25H enzyme regulate cholesterol normally [257,322,323]. Both 25OHC and 27OHC suppressed IgA production (a correlate of induction of innate immunity) whereas selective LXR ligands were inactive [257]. Synthetic LXR ligands were devoid of antiviral effects [309]. These studies argue that the protective effects of 25OHC are not mediated by LXR.

One possibility is that 25OHC is onward metabolized by the widely expressed oxysterol-metabolizing enzyme CYP7B1 [324] to 7α,25OHC. This molecule is a selective ligand of the G protein-coupled receptor EBI2, and experiments in knockout mice confirm that this route plays a role in modulating antigen-specific immunity. Knockout of either EBI2 or CH25H produced defects in activated B cell migration [322], and mice deficient in either CYP7B1 or CH25H display defective T cell-dependent responses [325]. However, this pathway is unlikely to explain the potent antiviral effects of 25OHC because only traces of 7α,25OHC were detected in the screen of Blanc *et al.*[309].

The specific receptor for 25OHC therefore remains unknown, although the molecule is known to bind with high affinity to oxysterol binding proteins OSBP1 and OSBP2 [326,327], and OSBP–sterol binding has been argued to play a specific regulatory role [328], notably in modulating OSBP subcellular localization [329]. 25OHC binding to OSBPs may interfere with essential intracellular targeting and delivery of pathogen components (specific intracellular trafficking pathways will differ between pathogens). OSBPs have been implicated in both AD and ATH [330,331]; oxysterol binding to OSBPs in macrophages is thought to play a direct role in atheromatous plaque formation [332] and macrophage expression of OSBP2 (OSBP-L1) enhances ATH in susceptible mice [331]. The estrogen receptor ERα remains a further contender as a target for 25OHC [333]. However, given the diverse variety of cellular binding sites for cholesterols [334], unraveling the specific molecular targets underlying the antiviral effects of 25OHC will be challenging.

#### 25OHC is implicated in both ATH and AD – the role of ACAT

In support of a role of CH25H enzyme in both diseases, GWAS studies have implicated the gene cluster cholesterol 25-hydroxylase, *CH25H* (10q23) – lipase A, lysosomal acid, cholesterol esterase, *LIPA* (10q23.2-q23.3) in both ATH [335,336] and AD [337,338]. The evolutionarily-conserved linkage between *CH25H* and *LIPA*, less than 20 kb in all mammalian species examined [339], is intriguing and suggestive because LIPA (lipase A, or cholesteryl ester hydrolase) is the primary enzyme responsible for de-esterification of cholesterol (see e.g., [340]).

Immunostimulation leads to induction of *CH25H* expression and local production of 25OHC. In AD, increasing expression of CH25H in temporal lobe regions of AD brain correlates with Braak (NFT) staging of disease progression [337]. No studies have been reported in human ATH, but elevated levels of 25OHC have been reported in lungs of patients with chronic obstructive pulmonary disease [341], another condition associated with chronic infection [342].

Chronic overexpression of CH25H is a powerful contender as the culprit for triggering disease pathology because, as first reported by Goldstein and Brown, it has been known for almost 40 years that 25OHC stimulates cholesterol esterification [316] (Figure 7B). Intracellular cholesterol esterification is catalyzed almost exclusively by ACAT (ACAT1/2) that adds a long-chain fatty acid to the cholesterol 3β-hydroxy group, and CH25H overexpression and 25OHC synthesis are known to promote ACAT activity, cholesteryl ester formation, and the generation of foam cells.

Compared to cholesterol itself, oxysterols are highly mobile, but once the 3β-hydroxy group has been attached to a long-chain fatty acid (principally oleic acid, C18; but also palmitic acid, C16 and other similar fatty acids) the molecule becomes insoluble and prone to aggregation. Although the major sterol in advanced ATH plaque appears to be 27OHC (>80% esterified) [303,304], and not apparently esters of 25OHC itself, this may be explained by the fact that 25OHC acts here, not as a substrate, but as an allosteric activator of intracellular esterification.

Work over many years, notably by Chang’s group, has revealed that ACAT enzymes contain two binding sites: (A) the allosteric regulatory site, and (B) the catalytic site. Once a sterol is bound to the A site the enzyme becomes highly active, with promiscuous substrate specificity for a wide range of sterols and even some steroids. Adding 25OHC to the culture medium caused a 20–60-fold increase in sterol esterification without change in enzyme content [317] (Figure 7B). Crucially, 25OHC is the most effective positive allosteric effector of ACAT; the enzyme is only poorly activated by close analogs such as 7-ketocholesterol, 6-ketocholestanol, 7α-OHC, cholate, or cholesterol itself [317,343] (Figure 7C). 25OHC activation of ACAT takes place in multiple cell types including macrophages [344] and neuronal cells [345]. In addition, it has been suggested that 25OHC drives intracellular redistribution of cholesterols to the endoplasmic reticulum [346], where ACAT is located; this could afford a second mechanism underpinning the enhancement of esterification. Therefore, by these routes 25OHC triggers the conversion of the intracellular pool of cholesterols (including 27OHC) into insoluble cholesteryl esters, and thus prevents their export (e.g., [347]). Induction of cholesterol esterification by 25OHC was recently confirmed [321].

In further confirmation of its key role, 25OHC has been shown to promote macrophage foam cell formation in mouse cell culture [318] (Figure 7D). It is interesting that 3 μM 25OHC is typically used to stimulate cholesterol esterification, whereas foam cell formation was reported to take place even at a 10-fold lower concentration [318]; ACAT is thus a further contender as a ‘receptor’ for 25OHC.

The data suggest that chronic overexpression of CH25H is causally associated with disease. In direct support, CH25H gene expression is repressed by transcription factor ATF3 – knockout of ATF3 in susceptible mice led to a marked increase in 25OHC levels and foam cell formation [318]. Ablation of CYP7B1 (that redirects 25OHC by conversion to the EBI2 ligand 7α,25OHC) increased ATH severity in mice, pointing to a specific predisposing effect of 25OHC itself [103]. Circumstantially, the finding that the expression of CYP7B1, that efficiently metabolizes 25OHC [324], is downregulated in AD brain [104] is consistent with the interpretation that excess 25OHC contributes to AD.

The finding that ACAT inhibition alleviates disease development in models of both ATH and AD (see earlier) confirms that ACAT-mediated sterol esterification, driven by 25OHC, is a key contributor to disease progression. This and other evidence argues that other atherosclerotic features (including disruption of endothelial/smooth muscle cell function and matrix calcification) are downstream consequences.

We suggest that chronic production of 25OHC in macrophages leads to ACAT-mediated sterol esterification, the accumulation of insoluble cholesteryl esters, foam cell formation, and vascular occlusion.

### Hypothesis: inflammation, macrophages, oxysterols, vasculature

**Figure 8:**
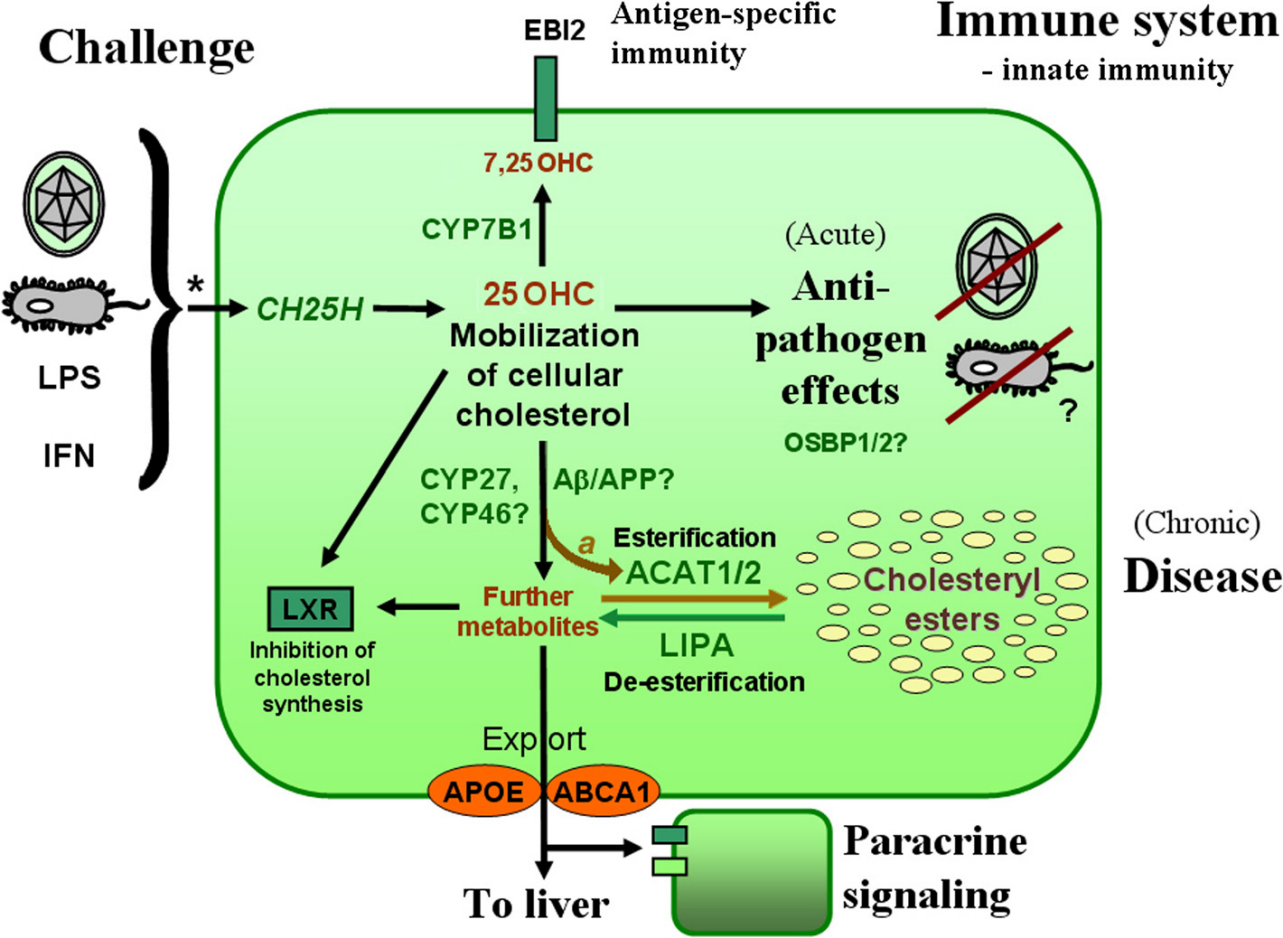
**The foam cell hub (simplified).** A model for disease development in ATH; similar processes are argued to operate in AD. Only key pathways are depicted. Challenge by infection or proinflammatory agents leads to induction of IFN signaling (*; both IFN-α and IFN-γ. For the specific pathways involved see [257,309] and references therein), upregulation of macrophage *CH25H* gene expression, and catalytic conversion of part of the cellular cholesterol pool to 25OHC. Acutely, this molecule exerts anti-pathogen effects via an unknown receptor. On chronic inflammatory exposure, hydroxylated cholesterols accumulate, are removed to insoluble intracellular inclusions by ACAT-mediated esterification (allosterically modulated by oxysterols, *a*), thereby leading to foam cell formation and overt disease. Oxidation by APP/Aβ may divert or enhance this process. APOE and ABCA1 are shown at the periphery to emphasize their role in export. Other key components from Table 1 (not shown in the figure) include LDLR, a trans-cellular receptor for cholesterol mobilization, and CLU, a protein with diverse functions in complement-mediated cell lysis, apoptosis, and lipid transport. Abbreviations: ABCA1, ATP-binding cassette, sub-family A (ABC1), member 1 (cholesterol efflux protein); ACAT, acyl-CoA cholesterol acyltransferase (also known as SOAT); APOE, apolipoprotein E; APP, amyloid-β (A4) precursor protein (Alzheimer precursor protein); CH25H, cholesterol 25-hydroxylase; CLU, clusterin (apolipoprotein J; complement cytolysis inhibitor); CYP27, cytochrome P450 27A1 (sterol 27-hydroxylase); CYP46, cytochrome P450 46A1 [cholesterol 24(S)-hydroxylase]; CYP7B1, cytochrome P450 7B1 (sterol and steroid 7α hydroxylase); EBI2, Epstein–Barr virus (EBV)-induced G protein-coupled receptor 2, IFN, interferon; LPS, lipopolysaccharide; LDLR, low-density lipoprotein receptor; LXR, liver X receptor; 25OHC, 25-hydroxycholesterol.

We propose a model in which immune stimulation by pathogens (or components/autoantigens) leads to local induction of CH25H in macrophages. Synthesis of 25OHC confers broad-spectrum inhibition of the growth of enveloped viruses, many of which preferentially target macrophages, and potentially modulates the growth of other pathogens including parasites and intracellular bacteria (Figure 8).

The downside of this anti-pathogen reaction is, by implication, that chronic production of 25OHC leads to accumulation of cholesteryl esters via the action of ACAT, the formation of fatty inclusions in macrophages, foam cell formation, and vascular occlusion (Figure 3). In turn, foam cells lose mobility and reside *in situ* for extended periods of time, themselves producing proinflammatory stimuli that amplify the pathology, leading to the formation of further atheromatous occlusions in the heart (ATH) or brain (AD) vasculature and overt disease.

#### The role of APP/Aβ

There is evidence that APP and Aβ, implicated in both AD and ATH, are themselves involved in oxysterol metabolism. In AD plaques Aβ colocalizes with deposits of APOE and cholesterol [348]. It was first reported that Aβ catalyzes the formation of 7β-OHC, and in this reaction Aβ was 200-fold more active than APP [349]. More recently evidence has emerged that Aβ has predominant cholesterol 3-oxidase activity [350,351], particularly in the presence of divalent cations such as Cu^2+^ (a known contributor to AD-like pathology in relevant models). Significantly elevated levels of 3-oxidized cholesterols were reported in brains of *APP*^AD^ mice and in postmortem brain tissue of AD patients [351]. 3- (or 7-) oxidation has potential to modulate the accumulation of cholesteryl esters in foam cells, although this remains to be demonstrated formally. APOE binding to Aβ [73] may also contribute but the role in disease is unknown.

However, the central involvement of Aβ remains contentious [166]. Aβ does not correlate precisely with clinical diagnosis of AD (reviewed in [352]), and the failure of Aβ immunotherapy to ameliorate AD pathology in clinical trials (not reviewed here) suggests that Aβ is not central, and is instead subservient to CH25H.

This has been confirmed via an unusual route. Papassotiropoulos *et al.*[337] report that, in large cohort of individuals with confirmed AD, specific *CH25H* haplotypes were associated with a complete absence of postmortem brain Aβ deposits, despite all other aspects of AD pathology. This argues that inflammation and CH25H activation are upstream to Aβ in the chain of events leading to AD.

The downstream induction of Aβ by infection/inflammation raises the question of whether Aβ might in fact be a protective (microbe-sequestrating or inactivating) response rather than a cause of pathology. In support, Aβ was shown to have potent antimicrobial properties, reducing the growth of *E. coli* by up to 200-fold *in vitro*, with activity against both Gram-positive and Gram-negative bacteria, and also against the fungus *Candida albicans*[353]. In the same study, brain homogenates from AD patients showed elevated antimicrobial activity versus control homogenates. It is not yet known whether Aβ-catalyzed sterol metabolism plays a role in this phenomenon. It is notable that reduction of Aβ in AD patients was associated with increased risk of infection [354] and, in both of two large clinical trials of anti-Aβ immunization in AD, 6% of patients developed encephalitis of unknown origin (reviewed in [355]), consistent with a potential defensive role of Aβ.

#### The role of APOE

Given the role of APOE in modulating disease (see above), by pathways that are only beginning to be understood, Robert Mahley argued that the diversification of APOE function, with three alleles prominent in the human population, has been driven by infectious disease. *APOE4* is probably the ancestral allele, and is most similar to homologs in other species [356], and subsequent selection pressure is thought to have led to the emergence of *APOE2* and *APOE3*[50].

The maintenance of multiple functionally different alleles in a population requires divergent selection – for which sickle-cell anemia affords the best precedent. Briefly, the hemoglobin S allele of β-globin is generally deleterious, but has a protective effect against malaria infection, and therefore in malaria-endemic areas the deleterious allele is maintained at high levels in the population [357].

This is paralleled by APOE, where APOE4 alleles are protective against some infectious diseases (e.g., malaria) but increase the proliferation of other pathogens (e.g., HSV-1, HIV) (see earlier). As stated by Mahley: ‘A cataclysmic event in human history driving the evolution of apoE4 to apoE3 to apoE2 could have been an infectious disease, such as the Great Plague, which killed 30–50% of Europeans in the 14th century, or smallpox’ [50].

We have argued here that chronic immune stimulation, leading to 25OHC production, contributes to the development of both ATH and AD. In both cases the *APOE4* allele is a major risk factor, and APOE4 can be associated with an increased load of specific pathogens that have been implicated in disease processes.

However, the exact mechanism is unknown. APOE4 is relatively poor at exporting cholesterols, and therefore would be expected to (i) increase intracellular immunosterols, thereby diminishing the proliferation of immunosterol-sensitive pathogens; and (ii) foster lipid droplet formation and vascular occlusion.

This is clearly an oversimplification, because 25OHC in cell culture inhibits several enveloped viruses (e.g., HSV-1 and HIV) whereas, *in vivo*, APOE4 increases their proliferation. This presents another conundrum that will only be resolved once we begin to understand the full spectrum of immunosterols {25OHC, 27OHC, 24(S)OHC, 24(S),25-epoxycholesterol, and others including cholesterol precursors and 7α derivatives}, their targets, and how they inhibit the proliferation of some pathogens while potentially enhancing others. Nevertheless, the evidence causally implicates CH25H in response to infection/inflammation as a triggering factor for cholesterol mobilization, esterification, and foam cell formation. Outstanding questions are summarized in Table 2.

**Table 2:** Outstanding questions

1	What is the molecular target for 25OHC?
2	Does the 'immunosterol' 25OHC also have anti-pathogen effects against bacteria and parasites?
3	Do other immunosterols, including 27OHC, 24(S)OHC, 24(S),25-epoxycholesterol, as well as derivatives of cholesterol precursors, operate by the same or different pathways? Do they confer protection to different types of pathogens?
4	How does APOE modulate infection? Is there a specific interaction between different polymorphic forms of APOE and oxysterols including 25OHC (or does it act via a partner such as APP/Aβ cholesterol oxidation)?
5	Is Aβ production a cause of disease, or is it a defense mechanism?
6	Does infection/inflammation in brain microglia also upregulate CH25H expression?
7	How do we explain the 'APOE paradox' – that *APOE4* is associated with highest risk of both AD and ATH, but *Apoe* knockout in mouse models accelerates ATH but delays AD development.
8	How do we explain the striking age-related increase in both AD and ATH incidence? Is it due to lifetime changes in circulating steroids/sterols?
9	Are other age-dependent diseases caused by a similar infection/inflammation/occlusion cascade?

### Concluding remarks

#### Brain versus body: why do some individuals develop AD, others ATH?

There are significant differences between brain and body; these could explain potential differences in the outcome of systemic infection and inflammation.

First, the brain differs from the body in cholesterol metabolism. In addition to being a net exporter of cholesterol, the CNS produces a brain-specific sterol, 24(S)OHC, known as ‘cerebrosterol’ [358]. The enzyme responsible, CYP46A1, is predominantly expressed in the brain [312] – most highly in areas affected by AD [359] – and polymorphisms in the gene cytochrome P450, family 46, subfamily A, polypeptide 1, *CYP46A1* (14q32.1), have been associated with risk of AD development (e.g. [360]). It is not known whether 24(S)OHC has specific immunoregulatory effects, but it is certainly plausible to suggest that that activity of CYP46 (also itself a 25-hydroxylase, see earlier) will impact upon the production and effects of 25-hydroxycholesterols, noting that 24(S),25-dihydroxycholesterol resolves to 24(S),25-epoxycholesterol, a further immunosterol with potent biological effects including inhibition of virus proliferation [309].

Second, some infectious agents home selectively to the brain and propagate therein. Examples include encephalitis viruses, poliovirus, rabies virus, and different members of the herpesvirus family. It is possible AD reflects infection and inflammation in association with the cerebrovasculature, whereas ATH is the result of infections propagating in the peripheral vasculature.

Third, the brain immune system differs from that in other tissues. The brain contains its own specialized macrophage-like cells, the microglia, that share with macrophages the properties of self-renewal, mobility, cytokine- and chemokine-responsiveness, antigen presentation, and phagocytosis, although the brain contains both typical macrophages and microglia. Brain microglia could potentially contribute to AD development, but the evidence is inconclusive.

Fourth, microtubule-associated protein Tau is particularly abundant in CNS neurons, and AD is associated with intracellular aggregates (NFT) of Tau. APOE3 binds avidly to Tau whereas APOE4 shows no significant binding. This interaction may modulate AD development [361] but only make a minor contribution to ATH.

#### Causes, cures, and age-dependence

In both ATH and AD we see central involvement of vascular pathology, clinical association with common predisposing genes/alleles, association with infection, disease enhancement by immune stimulation, the central role of bone marrow-derived cells, principally macrophages, the involvement of Aβ, drug overlap, and cholesterol involvement. These findings suggest that the two diseases share a common pathoetiology. A clear hypothesis is emerging in which chronic infection/inflammation in the vasculature leads, in the short term, to blockade of pathogen proliferation – but in the longer term to 25OHC-driven fatty droplet accumulation and vascular occlusion. Is a battle being played out in our vasculature, where the accumulation of cholesteryl esters, precipitating ATH or AD, is the price of surviving the onslaught of an invading pathogen?

Selective inhibitors of CH25H and/or ACAT may therefore have clinical potential in both diseases; indeed, some widely-available dietary components (with likely activity in these pathways) are under investigation as possible protective agents. Conversely, preventive measures against pathogen infection (e.g., vaccination) are unlikely to be productive, not only because of the diversity of potentially causal agents but also because, for several pathogens, a substantial proportion of the population is already positive from childhood onwards.

In addition, remarkably, in some cases early infection could even be beneficial. Barnton *et al.*[362] report that latent infection of mice with herpes viruses (mouse gammaherpesvirus 68 or CMV) confers resistance to bacterial infection, with a 100–1000-fold reduction in the replication of *Listeria monocytogenes* or *Yersinia pestis* (but without effect on a viral pathogen, West Nile virus). Resistance was not antigen-specific, and required chronic IFN-γ production, suggesting that similar pathways (Figure 8) may be exploited by latent viruses – providing a first indication that bifurcation of immunosterol pathways might potentially foster one pathogen at the expense of another.

The striking age-dependence of disease is not understood. For many pathogens, infections are acquired in early childhood and remain stable, but for others there is a clear age-related increase in seropositivity rates (not reviewed). A recent exciting development is that macrophages show an age-related decline in the expression of the key cholesterol efflux protein, ABCA1 [363], implicated in both ATH and AD (see earlier and Table 1). However, given that age-related phenomena are controlled systemically rather than being cell-intrinsic (e.g., [364]), a more attractive hypothesis might be that the well-documented lifetime declines in tissue levels of steroidal molecules such as 7-dehydrocholesterol (a precursor of vitamin D), allopregnenolone, and dehydroepiandrosterone (DHEA), that interact with the self-same sterol pathways discussed here, contribute to disease processes. Indeed, protective effects of vitamin D and DHEA in both ATH and AD models have been reported; lifetime changes in androgens and estrogens may also play a role.

#### Ill or just old?

This debate has highlighted many lines of evidence that two major age-related diseases are likely to be precipitated by infection and inflammation, and are not merely a consequence of age *per se*. However – in the terms of Izaks and Westendorp [1] – infection/inflammation is a ‘component cause’, but not a ‘sufficient cause’. There are undoubtedly good reasons to suspect that a systemic age-related component so far unidentified contributes to both diseases.

This is an exciting area of research, and one hopes that the next years will bring a new understanding of why some individuals develop AD, others ATH, whereas many avoid both conditions. There are implications for drug development, and drugs with moderate efficacy in either ATH or AD warrant testing in the other disorder. Further research will be necessary to unravel the molecular underpinnings that link infection, oxysterols, and the age-relatedness of these two major diseases.

## Summary

1. We propose that ATH and AD share a common infectious/inflammatory pathoetiology.

2. Both have similar age-dependence, vascular pathology, genetic underpinnings including the central role of *APOE*, Aβ involvement, and association with infection. Both diseases are dependent on bone marrow-derived cells, principally macrophages, and show overlapping drug responsiveness.

3. We postulate a causal mechanism. (i) Infection and inflammation, notably in macrophages associated with the vasculature, induce the expression of CH25H. (ii) Acutely, the enzymatic product 25OHC provides protection against pathogen propagation; chronically, macrophage 25OHC activates ACAT, leading to cholesterol esterification, lipid droplet formation, and ultimately vascular occlusion.

4. We infer that, in individuals with ATH or AD, they are neither merely ‘Ill’ nor ‘Old’, but instead both acquired infection/inflammation and endocrine aging are likely to play a joint role in causing these age-related diseases.

## Competing interests

The authors declare that they have no competing interest.

## Authors’ contributions

RL was responsible for the concept, SS researched genetic underpinnings and links to infection, and YK investigated links with vasculature occlusion. All authors contributed to writing the manuscript, and all have read and approved the final version.

## Pre-publication history

The pre-publication history for this paper can be accessed here:

http://www.biomedcentral.com/1471-2318/14/36/prepub
